# Integrated genomic analysis reveals mutated *ELF3* as a potential gallbladder cancer vaccine candidate

**DOI:** 10.1038/s41467-020-17880-4

**Published:** 2020-08-24

**Authors:** Akhilesh Pandey, Eric W. Stawiski, Steffen Durinck, Harsha Gowda, Leonard D. Goldstein, Mustafa A. Barbhuiya, Markus S. Schröder, Sreelakshmi K. Sreenivasamurthy, Sun-Whe Kim, Sameer Phalke, Kushal Suryamohan, Kayla Lee, Papia Chakraborty, Vasumathi Kode, Xiaoshan Shi, Aditi Chatterjee, Keshava Datta, Aafaque A. Khan, Tejaswini Subbannayya, Jing Wang, Subhra Chaudhuri, Sanjiv Gupta, Braj Raj Shrivastav, Bijay S. Jaiswal, Satish S. Poojary, Shushruta Bhunia, Patricia Garcia, Carolina Bizama, Lorena Rosa, Wooil Kwon, Hongbeom Kim, Youngmin Han, Thakur Deen Yadav, Vedam L. Ramprasad, Amitabha Chaudhuri, Zora Modrusan, Juan Carlos Roa, Pramod Kumar Tiwari, Jin-Young Jang, Somasekar Seshagiri

**Affiliations:** 1 Institute of Bioinformatics, Bangalore, Karnataka 560066 India; 2 Manipal Academy of Higher Education (MAHE), Manipal, Karnataka 576104 India; 3 Center for Individualized Medicine and Department of Laboratory Medicine and Pathology, Mayo Clinic, Rochester, MN 55905 USA; 4 Bioinformatics and Computational Biology Department, Genentech Inc, South San Francisco, CA 94080 USA; 5 Molecular Biology Department, Genentech Inc., South San Francisco, CA 94080 USA; 6 Research and Development Department, MedGenome Inc, Foster City, CA 94404 USA; 7 QIMR Berghofer Medical Research Institute, Brisbane, QLD 4006 Australia; 8 Department of Pathology and Laboratory Medicine, Pennsylvania State University College of Medicine, Hershey, PA 17033 USA; 9 SciGenom Labs, Cochin, Kerala 682037 India; 10 Department of Surgery, Division of Hepatobiliary and Pancreatic Surgery, Seoul National University Hospital, Seoul National University College of Medicine, Seoul, 08826 South Korea; 11 Research and Development Department, MedGenome Labs Pvt. Ltd., Bangalore, Karnataka 560099 India; 12 Department of Pathology, Cancer Hospital and Research Institute, Gwalior, Madhya Pradesh 474009 India; 13 Department of Surgical Oncology, Cancer Hospital and Research Institute, Gwalior, Madhya Pradesh 474009 India; 14 Jiwaji University, Gwalior, Madhya Pradesh 474011 India; 15 Department of Pathology, Millennium Institute on Immunology and Immunotherapy, School of Medicine, Pontificia Universidad Católica de Chile, Santiago, Chile; 16 Applied Molecular and Cellular Biology PhD Program Universidad De la Frontera, Temuco, Chile; 17 Department of Surgery, Postgraduate Institute of Medical Education and Research, Chandigarh, 160012 India; 18 SciGenom Research Foundation, 3rd Floor, Narayana Nethralaya Building, Narayana Health City, #258/A, Bommasandra, Hosur Road, Bangalore, Karnataka 560099 India

**Keywords:** Cancer genomics, Immunotherapy

## Abstract

Gallbladder cancer (GBC) is an aggressive gastrointestinal malignancy with no approved targeted therapy. Here, we analyze exomes (*n* = 160), transcriptomes (*n* = 115), and low pass whole genomes (*n* = 146) from 167 gallbladder cancers (GBCs) from patients in Korea, India and Chile. In addition, we also sequence samples from 39 GBC high-risk patients and detect evidence of early cancer-related genomic lesions. Among the several significantly mutated genes not previously linked to GBC are ETS domain genes *ELF3* and *EHF*, *CTNNB1*, *APC*, *NSD1*, *KAT8*, *STK11* and *NFE2L2*. A majority of *ELF3* alterations are frame-shift mutations that result in several cancer-specific neoantigens that activate T-cells indicating that they are cancer vaccine candidates. In addition, we identify recurrent alterations in KEAP1/NFE2L2 and WNT pathway in GBC. Taken together, these define multiple targetable therapeutic interventions opportunities for GBC treatment and management.

## Abstract (web summary)

Gallbladder cancer incidence shows characteristic geographic patterns. Here the authors perform a genomic analysis of gallbladder cancers in patients from countries with high incidence (South Korea, India and Chile) and identify ELF3 and other significantly mutated genes not previously associated with gallbladder cancer.

## Introduction

The gallbladder is an important part of the biliary tract system. Gallbladder cancer (GBC) is the most common of the biliary tract cancers^1^. GBC is a highly fatal malignancy with median survival of <1 year^2–4^. This is primarily due to non-specificity of symptoms during initial stages of the disease with patients generally presenting at an advanced stage of the cancer. GBC often occurs in the setting of gallstones (cholelithiasis) or chronic inflammation (cholecystitis) and is typically detected incidentally in patients undergoing treatment for these conditions^1^. The diagnosis is also confounded by the anatomic position of the gallbladder and the non-specificity of the symptoms during the initial stages of the disease^1^.

GBC is the 20th most common cancer worldwide with an estimated 178,100 new cases diagnosed annually^4^ (http://globocan.iarc.fr). In 2020, in the United States, an estimated 11,980 new cases and 4,090 deaths due to gallbladder cancer is expected (https://www.cancer.net/cancer-types/gallbladder-cancer). In contrast to the general population in the United States where GBC incidence is low, it is a more common gastrointestinal malignancy in both Southwestern Native Americans and in Mexican Americans^4^. Incidence of GBC is highest in South American countries that include Chile, Bolivia, and Ecuador and Asian countries such as Korea, India, Pakistan and Japan. Interestingly, GBC incidence is lowest in Africa^1,4^ (http://globocan.iarc.fr). In addition to race, GBC incidence increases with age and women are affected two to six times more often than men^4^. Other recognized risk factors for GBC development include the occurrence of gallstones and *Salmonella* infection^4^.

Previous molecular studies on GBC have focused on the assessment of mutations in few candidate genes such as *TP53* and *KRAS*^1^. Recent exome sequencing of nine GBC samples of Caucasian origin identified *TP53* as a significantly mutated GBC gene^5^. Another study examined exomes from 32 GBC samples of Chinese origin and identified *TP53*, *KRAS*, and *ERBB3* as significantly mutated GBC genes^6^. Further, exome sequencing and analysis of 28 GBC patients of Japanese origin and targeted sequencing of 51 samples of Chinese origin identified alterations in ERBB family members^7^. A recent follow-up study^6^ reported exome data from an additional 125 additional Chinese patients linking frequent *ERBB2/3* mutation and upregulation of *PD-L1* in GBC^8^. A recent study reported exome sequencing of 16 GBC samples of Japanese origin and targeted sequencing of 30 GBC samples that included 26 Italian and 4 Japanese patients^9^.

Given that GBC incidence shows strong geographic variation, in this study we perform a comprehensive analysis of 167 GBCs that includes patients from three geographically different regions namely, South Korea (*n* = 94), India (*n* = 64) and Chile (*n* = 9). We analyze 167 tumors from three geographically distinct parts of the world and identify *ELF3* to be a significantly mutated gene. Given our large sample size, we find several previously unreported significantly mutated GBC genes. The altered genes include *TP53, ELF3, ERBB3, CTNNB1, ARID2, CDNK2A, STK11, SMAD4, ARID1A, EHF, KRAS, NFE2L2, PIK3CA*, and *PSIP1*. We further identify a class of mismatch-repair-deficient gallbladder cancers with elevated mutation rates which are likely candidates for immunotherapy. The ELF3 mutations are predominantly frame-shift alterations that result in several neoantigens that are able to activate CD8+ T-cells, confirming them as potential cancer vaccine candidates.

## Results

### Genomic analysis of GBC samples

We have performed a comprehensive genomic analysis of 213 samples that included 167 gallbladder (GBC) primary tumors, 7 GBC cell lines, 23 gallbladder tissue from cholecystitis cases (cholecystitis), 14 gallbladder tissue from gall stone cases (stone), and 2 gallbladder polyps (polyp). Overall, we have obtained whole exome (WES) from 206 samples (160 GBC, 23 cholecystitis, 14 stone, 2 polyps and 7 cell lines), RNA-seq from 120 samples (115 GBC and 5 cell lines) and low pass (<5x) whole-genome sequence (WGS) data from 184 samples (146 GBC, 15 cholecystitis, 14 stone, 2 polyps and 7 cell lines; Supplementary Table 1, Supplementary Data 1, and Supplementary Fig. 1).

For 98 of the 167 GBC cases in this study we have obtained exome, RNA-seq and low pass WGS data, making this a comprehensive GBC genomic data set (Supplementary Table 1, Supplementary Data 1 and Supplementary Fig. 1).

### GBC mutational profile

**Fig. 1:**
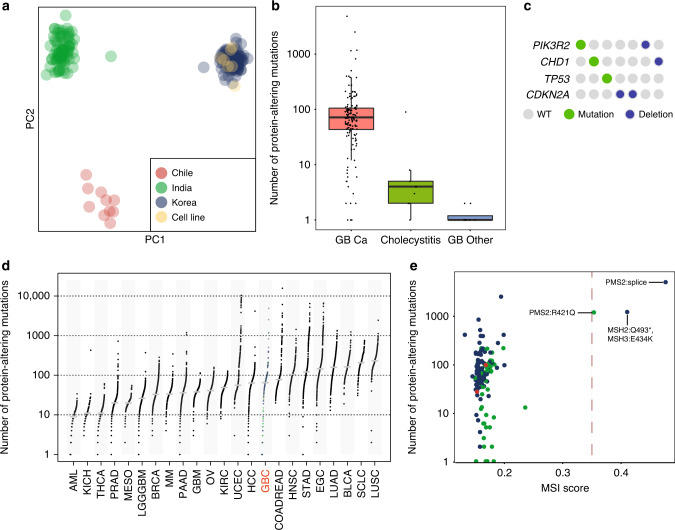
Genetic variation in GBC and non-GBC samples. **a** Principal Component Analysis of germline variants of GBC and non-GBC samples colored based on country of origin. **b** Box plot depicting the number of protein-altering mutations in GBC (*n* = 148), Cholecystitis (*n* = 9) and GB-other (*n* = 8) samples containing mutations (1 or more). Boxes indicate the interquartile range (IQR); center line, median; whiskers, lowest and highest values within 1.5x IQR from the first and third quartiles, respectively. **c** Quilt plot showing mutations in key cancer-associated genes in cholecystitis samples. Each column represents a sample. **d** Number of protein-altering somatic mutations in GBC compared to mesothelioma^66^ and 21 other cancer types. **e** MSI score determined by MANTIS plotted against the total number of protein-altering mutations for each GBC sample. Samples are colored based on country of origin as in panel **a**. MMR gene mutations identified are indicated next to the MSI positive samples.

We obtained WES data on 160 GBC (152 patient-matched paired tumor/normal GBC and 8 unpaired GBC) from India (60), Korea (91), and Chile (9). Also, we obtained WES data for 7 GBC cell lines. In addition, we surveyed pre-cancerous gallbladder tissue samples from 23 cholecystitis cases, 14 gallbladder stones, and 2 gallbladder polyps by WES (Supplementary Table 2 and Supplementary Data 2–8). Samples were sequenced at an average coverage of 93x and tumor/normal relationships were confirmed using exome sequence data (Supplementary Data 2 and Methods). Principal component analysis (PCA) using the germline variants from the matched normal revealed that the samples clustered into groups based on their population of origin (Fig. 1a and Supplementary Fig. 2). The six-cell lines derived from patients of Japanese ancestry and one from a Korean patient clustered with the Korean samples consistent with a north east-Asian genetic profile (Fig. 1a and Supplementary Fig. 2). Amongst the patient-matched paired tumor-normal GBC samples, a total of 21,439 protein-altering somatic mutations were identified, including 17,475 missense, 1215 nonsense, 26 stop loss, 22 start lost, 419 essential splice-site mutations, and 2282 indels (Supplementary Table 2 and Supplementary Fig. 3). A majority of the mutations (92%; 19,757/21,439) were novel and were not reported in COSMIC.v70^10^ (Supplementary Table 2). Using RNA-seq data, we confirmed the expression of 8,706 protein-altering somatic variants identified by WES (Methods, Supplementary Table 2, Supplementary Data 3 and 4).

Both cholecystitis and gallstones (cholelithiasis) are believed to lead to precancerous lesions by inducing dysplastic changes in the pathogenesis of GBC^1^. We sequenced tissue from areas surrounding the inflamed sites in the gallbladder tissue from patients with chronic cholecystitis (*n* = 23), or gallbladder stones (*n* = 14) and gallbladder polyps (*n* = 2) together designated ‘GB-other’. We found significantly fewer somatic protein-altering mutations with a median of 1 (range 0–90) for cholecystitis and a median of 0.5 (range 0–2) for GB-other compared to a median of 65 (range 0–4,867) in GBCs (Fig. 1b and Supplementary Data 5). We next looked for cancer-associated somatic alterations using a combination of exome and low pass WGS data and found mutations or copy loss in 30% (7/23) of cholecystitis samples (Fig. 1c). The alterations included *PIK3R2*, *CHD1*, *TP53*, and *CDKN2A*. Consistent with this, we found alterations in *TP53*, *CDKN2A*, *PIK3R2*, and *CHD1* in GBC (Supplementary Data 3 and Supplementary Fig. 4). Previously, *TP53* and *CDKN2A* have been implicated as GBC drivers^5–7^. Though not previously implicated in GBC, *CHD1* is a known gastric cancer driver and *PIK3R2* is frequently mutated in endometrial cancers^11–13^. In contrast to cholecystitis samples, somatic alterations in the ‘GBC-other’ group were not detected.

We found the median mutation rate in GBC to be between that of hepatocellular carcinoma (HCC) and colorectal cancers (COADREAD; Fig. 1d). However, we found three outlier samples with very high mutation burden. We tested these samples for microsatellite instability (MSI) using the MANTIS^14^ (see Methods) and found that they were positive for MSI. Consistent with this, all the three samples had a high mutation burden (>1000 protein-altering mutations) (Fig. 1e). We confirmed that these samples carried deleterious mutations in known mismatch repair genes (Fig. 1e). Interestingly, the outlier mutational status of the MSI positive samples was similar to that of mismatch repair-deficient colorectal cancer (COADREAD), endometrial carcinoma (UCEC), and stomach adenocarcinoma (STAD) (Fig. 1e).

To understand the mutational processes that contribute to the development of GBC we identified and cataloged the possible 96 base substitution types taking into account the possible eight base pair somatic changes (C > T, T > C, C > A, C > G, T > A, and T > G) and the flanking 5’ and 3’ base context, as previously described^15^. The substitution frequencies between GBC samples from India, Chile and Korea were similar and was consistent with the frequency pattern observed in a set of 29 GBCs from Japan^7^ (Supplementary Fig. 5). Using non-negative matrix factorization, as described previously (see Methods), we identified six prominent mutational signatures (Supplementary Figs. 5 and 6a, b). The strongest correlates included age (C > T mutations at NpCpG sites), an APOBEC signature (dominated by C > T and C > G mutations at TpCpN sites), and an MSI signature. These findings are consistent with previously identified processes in gallbladder and ampullary cancers^7,16,17^. When we compared the mutation profile to 8 other cancer types, we found it clustered most closely to head and neck squamous cell carcinoma (Supplementary Fig. 7).

### Mutated genes and their significance in GBC

Exome sequencing identified protein-altering somatic mutations in 10,224 genes and of these 4750 (46%) were mutated in at least two patients. We found recurrent mutations in 102 chromatin-modifying genes, including *NSD1*, *ARID1A, SETD2*, and *PBRM1*, 187 protein kinases, including *TTN, ERBB2, ERBB3, STK11*, and *LATS1*, and 73 G-protein coupled receptors including *CELSR1/2/3, CHRM3,* and *GRM1*. Using Polyphen^18^, we found that 55% (11,716/21,439) of protein-altering mutations were predicted to be deleterious or high impact mutations. In contrast, only 11% (233,524/2,122,090) of the protein-altering germline variants from this study were predicted to have a functional impact.

**Fig. 2:**
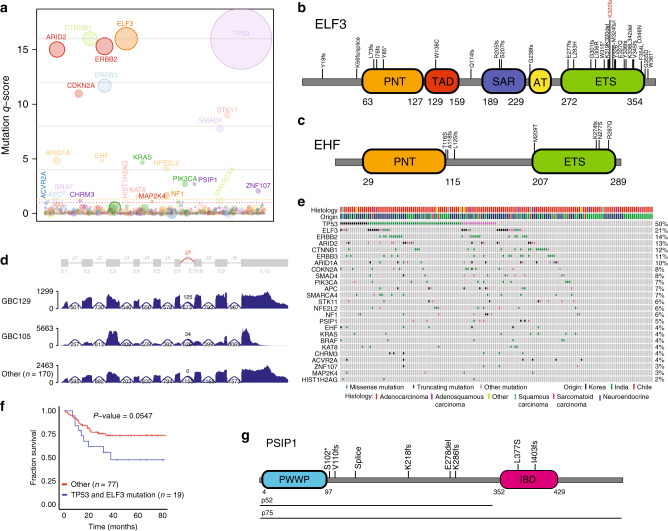
Significantly mutated genes in GBC. **a** Plot showing the significantly mutated GBC genes and their *q*-score. Each gene is represented as a circle, where the size of the circle is proportional to the observed mutation frequency. Genes are arranged along the *x* axis in alphabetical order. Significant *q*-score genes with FDR < 0.1 are indicated. Dotted orange line, FDR = 0.05, dotted red line, FDR = 0.1. Schematics showing alterations in ETS family members (**b**) ELF3 and (**c**) EHF. **d** ELF3 splicing defects observed in two GBC samples. **e** Plot representing mutations observed in SMG genes across the GBC samples. Each column represents a sample. **f** Kaplan–Meier survival plot of patients with tumor double positive for ELF3 and TP53 vs others. Log-rank test *p* values are presented for each group. **g** Schematic showing alterations observed in PSIP1 in GBCs.

We assessed the mutated genes for their significance using a q-score metric^19^. Our analysis identified 25 significantly mutated GBC genes that included *CTNNB1, ELF3, TP53, ERBB2, ARID2, ERBB3, STK11, CDKN2A, SMAD4, ARID1A, KRAS, EHF, PIK3CA, BRAF, ACVR2A, PSIP1, NFE2L2, CHRM3, ZNF107, SMARCA4, APC, NF1, KAT8, MAP2K4* and *HIST1H2AG* (Fig. 2a; *q*-score ≥ 1.1; FDR ≤ 8%; Supplementary Table 2 and Supplementary Fig. 8a–d). This list includes well-known oncogenes, *CTNNB1, ERBB2, ERBB3, KRAS, PIK3CA, BRAF*, and *NFE2L2*, tumor suppressors, *TP53, ARID2, STK11, CDKN2A, SMAD4, SMARCA4, ARID1A, APC, NF1*, and *MAP2K4*, and less well-established cancer-associated genes such as *ELF3, EHF, ACVR2A, PSIP1, CHRM3, HIST1H2AG, KAT8*, and *ZNF107*. Previous studies on GBC reported *TP53, KRAS* and *ERBB3* as significantly mutated GBC genes (SMG)^6^, though low-frequency mutations were observed in other SMG GBC genes reported in this study^6,7,20^.

The ELF3, ETS-domain transcription factor, identified as significantly mutated gallbladder cancer gene was altered in 21% of samples (34/160) (Fig. 2b). Previously, *ELF3* was reported as a frequently mutated gene in biliary tract (3–9.5%) and ampullary carcinomas (15%)^6,7,9,16,17^. *ELF3* is also known to be mutated in cervical adenocarcinomas (13%)^21^, bladder cancers (8%)^22^, gastric cancers (4%)^23^, and colorectal cancers (3%)^24,25^. In addition to *ELF3, EHF*^26^, another member of the ETS transcription factor subfamily, was also found significantly mutated  in 4% of the GBC samples (7/160) (Fig. 2c).

A majority of *ELF3* mutations we observed were frame-shift, stop gained and essential splice-site mutations (73% 27/37 mutations) and they clustered in the C-terminal ETS-domain (Fig. 2b). In addition, two essential splice-site mutations that result in an RNA transcript that codes for a truncated *ELF3* (Fig. 2d) were found. Interestingly, *ELF3* mutations were more frequent in Korean (31% 28/91) and Chilean patients (22% 2/9) compared to GBC patients from India (7% 4/60; *p* value 0.0003 India vs non-Indian; Fisher’s exact test).

We observed that the *ELF3* mutations co-occurred significantly with *TP53* mutations (*p* value = 0.01; Fig. 2e). Patients carrying both *ELF3* and *TP53* mutations had a worse overall survival that showed a trend towards significance (*p* value 0.0547; Fig. 2f) as opposed to no difference in survival in individuals with mutations in just either one of the genes (Supplementary Fig. 8d).

In addition to the WNT pathway genes *CTNNB1* and *APC*, the significantly mutated genes included the chromatin-modifying gene *KAT8*, tumor suppressor *STK11*, oncogene *NFE2L2*, and *ZNF107* that codes for a zinc finger protein. In addition, *ACVR2A*, a serine-threonine kinase and a member of the TGF-beta superfamily was also found to be mutated. The chromatin-associated protein gene, *PSIP1*, also showed a distinct mutation pattern with 5 of the 6 mutations (4% of samples) showing high impact frame-shift mutations (*n* = 4) or a premature stop codon (*n* = 1). These mutations preserved the PSIP1 H3K36me3 interacting ‘Pro-Trp-Trp-Pro’ (PWWP) domain while leading to the loss of the region coding for the C-terminal ‘integrase binding’ (IBD) domain (Fig. 2f). Interestingly, p52 PISP1 is a well-characterized isoform that lacks the IBD and has been associated with transcriptional activation and alternative splicing^27^ and its relevance in GBC requires further investigation. Another significantly mutated gene, *CHRM3* encodes a GPCR muscarinic cholinergic receptor. Interestingly, *CHRM3* was recently shown to mediate gallbladder contraction through a voltage-gated Ca2+ channel^28^. However, the exact relevance of the *CHRM3* mutations in GBC needs further studies.

Recurrence of somatic mutations is an indication of its cancer relevance^29^. We examined our data for recurrent hotspot mutations and found 89 hotspot mutations across 73 genes (see Methods; Supplementary Data 8). Included in the genes with hotspots were 11 significantly mutated GBC genes. Genes with most hotspots included *TP53* (11 in 53 samples), *ERBB2* (3 in 18 samples), *CTNNB1* (3 in 16 samples), and *ELF3* (3 in 9 samples). The *ELF3* hotspot mutations included frame-shift mutations at codons 55 (2 samples), 320 (5 samples), and 324 (2 samples, including 1 missense).

To further understand specific mutation patterns, we performed a meta-analysis by comparing all the somatic mutations identified against a list of high confidence hotspot mutations identified in a comprehensive pan-cancer analysis^30^. We identified 65 meta-hotspot mutations across 22 genes (Supplementary Data 9). The most common genes identified in GBC were *TP53* (22 in 40 samples), *ERBB2* (5 in 19 samples), and *CTNNB1* (5 in 18 samples, primarily concentrated around codons 32–45). Amongst the most common mutations across all cancers that also occurred in GBCs were KRAS G12/G13 (5 samples), PIK3CA H1047/E545/E542 (6 samples) and NRAS Q61 (1 sample). Other mutations of interest included ERBB3 (2 meta-hotspots in 7 samples) carrying activating mutations at V104 and D297^31^. Interestingly, the four BRAF meta-hotspots, G466A (1), G469V (1), D594G (2), G596R (1), observed in 5 samples did not involve the canonical V600 codon. The NFE2L2 (5 meta-hotspots in 5 samples) mutations were primarily concentrated around amino acid positions 29–34. We also found samples containing CDK4 R24C, BCL2L12 R18W, RAC1 A178E, and XPO1 E571K mutations, previously reported in other tumor types^30^.

### Splicing, expression and copy number alterations in GBC

We performed t-SNE analysis of RNA-seq data from 115 GBC and identified two main clusters designated as cluster A and B (Supplementary Fig. 9). Cluster A is characterized by high expression of mitochondrial genes and also showed high levels of apoptosis-related gene such as *BAX, BAD, FASTK*, and *NOXA1*. Further, expression of *PTEN, SMAD4, NF1*, and *NF2* was low in cluster A compared to samples in cluster B. Interestingly, samples in cluster B had marked upregulation of oncogenes such as *BRAF, KRAS*, and *CBL*. Several histone encoding genes were also upregulated in cluster B. We also found upregulation of *NPAT* and *GONL4* in cluster B. *NPAT* is a key co-activator of histone transcription and *GON4L* is involved in biogenesis of the histone locus bodies and a known *NPAT* binding partner^32^. Additionally, cell cycle regulators such as *SPDYE1* and *SPDYE2* were also highly expressed in cluster B. Also upregulated in cluster B were transcripts for TP53 modulators *ATM* and *MDM4*.

We performed de novo prediction of splice variants from 115 GBCs and 4 cholecystitis samples to identify tumor-specific splicing events. We considered 835 candidate cancer-associated genes and filtered out splice variants expressed in a dataset of 9155 normal samples^33^. We identified 62 candidate protein-altering splice variants in 24 samples. They included recurrent variants in *ELF3* (*n* = 2) (Fig. 2d), an alteration each in *KEAP1* and *NFE2L2* (exon 2 deletion) (Supplementary Data 10). The exon 2 deletion in *NFE2L2* variant was previously described in squamous cell carcinoma^34^ and is known to result in the loss of interaction with the negative regulator KEAP1, NFE2L2 stabilization, induction of a NFE2L2 transcriptional response, and KEAP1/NFE2L2 pathway dependence.

We performed copy number analysis using low pass WGS data from 146 GBC samples. *ERBB2* was frequently amplified in 13% (19/146) of the GBCs. We confirmed overexpression of *ERBB2* in 68% (13/19) of the GBCs with amplification (Supplementary Fig. 10). Furthermore, we found one sample (GBC138) with *EGFR* amplification and corresponding increased *EGFR* expression (Supplementary Fig. 10). We also found amplification of *MET* (GBC061), *KRAS* (GBC009), and *NRAS* (GBC001). These genes also showed elevated expression in the corresponding samples (Supplementary Fig. 10). Chromosome 12 showed a distinct recurrent amplification in 6 samples involving *YEATS4, RAB3IP, a*nd *FRS2*. We found expression of these genes to be elevated in these samples (Supplementary Figs. 11 and 12). Among genes that showed copy loss were *CDKN2A/B* (14/146), *SMAD4* (3/146), *FHIT* (11/146), *BAP1* (7/146). *PBRM1* (3/146) and *PTEN* (2/146) and this correlated with lower expression of these genes in the corresponding samples (Supplementary Fig. 13).

### Gene fusions in GBC

Analysis of RNA-seq data identified 23 gene fusion events in our GBCs (Supplementary Table 3). In one sample, we found a fusion involving *PTPRK* and *RSPO*  (Supplementary Fig. 14) that led to overexpression of *RSPO3*. This gene fusion product is known to promote and potentiate WNT signaling^24^. Also, we found a recurrent fusion involving two patient tumors where exon 1 of *GSK3A* was fused in-frame to exon 3 of *CDC42EP1*, resulting in a transcript coding for GSK3A lacking the kinase domain (Supplementary Fig. 15a). A gene fusion involving *PTEN* and *LIPA* **(***LIPA*-PTEN) leading to removal of the sequence encoding the PTEN Tensin C2 domain was identified in a patient tumor (Supplementary Fig. 15b). Another GBC sample carried an *SLC12A7-TERT* fusion that resulted in overexpression of *TERT* (Supplementary Fig. 15c). We also found an in-frame *GRB7-LASP1* fusion resulting in elevated *LASP1* expression (Supplementary Fig. 15). Upregulated *LASP1* has been linked to malignant phenotype in cholangiocarcinoma^35^.

### Mutated ELF3 neoantigen peptides activate CD8+ T-cells

**Fig. 3:**
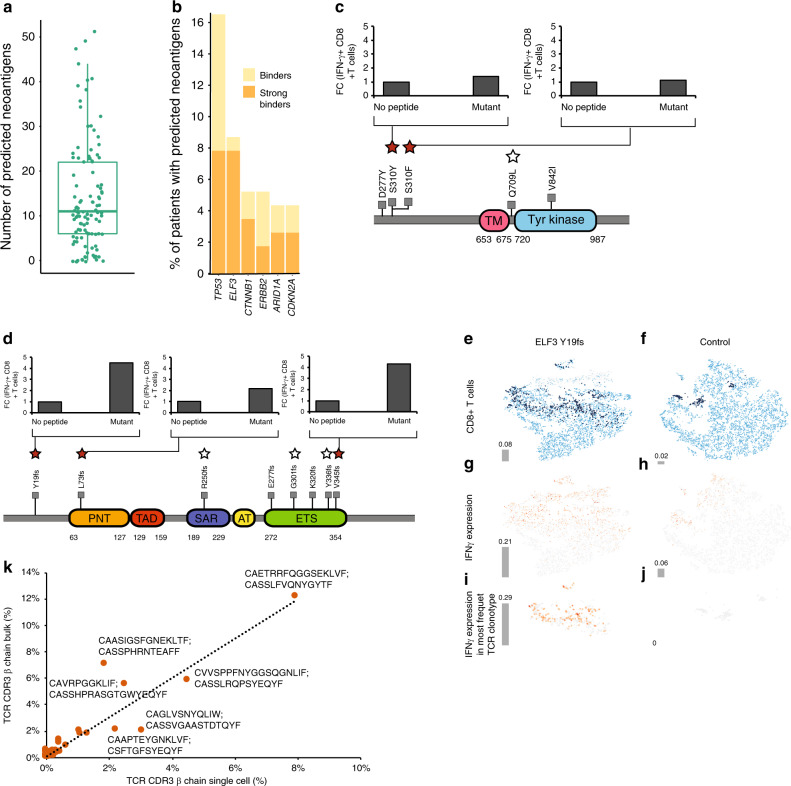
Neoantigens in GBC. **a** Box plot of neoantigens predicted in GBC samples (*n* = 110). Boxes indicate the interquartile range (IQR); center line, median; whiskers, lowest and highest values within 1.5x IQR from the first and third quartiles, respectively. **b** Stacked bar plot of genes with the highest number of predicted neoantigens plotted as a percentage of total number of patient tumors with a mutation in that gene (*n* = 43).  Peptides with predicted  MHC I affinty of ≤500 nm (yellow)  and <500 nm (strong binder; orange) are shown in. Schematic of ERBB2 (**c**) or ELF3 (**d**) neoantigen generating mutations and CD8+ T-cell activation measured by INF-γ expression level for the indicated mutant peptides. Stars above the mutation represent peptides tested and solid stars represent CD8+ T-cell activating peptides. t-SNE plots based of single-cell RNA/TCR-seq data for CD8+ T-cells (light blue) co-cultured with dendritic cells expressing either the ELF3 Y19fs minigene (**e**) or control vector (**f**). Dark blue represents the most abundant αβ CD8 TCR clone and gray is non-CD8+ T-cells. IFN-γ expression in the total cell population primarily enriched in CD8+ T-cells in the ELF3 Y19fs minigene (**g**) or control vector (**h**) sample. **i, j** IFN-γ expression observed in the expanded CD8+ T-cells identified by TCR sequence analysis. **k** A scatter plot of correlation between the most frequently represented TCR clones identified by the TCRβ chain sequence in the single-cell and PMBC CD8+ T-cell activation assay (see Methods). The TCR α and β chain CDR3 sequence of the top six most clonally expanded T-cell clones are shown.

Recent advances in cancer immunotherapy have led to impressive survival benefit for patients in some cancers^36,37^. Understanding the neoantigens arising from somatic mutations and the composition of tumor immune microenvironment will provide opportunities for immunotherapy in GBC. With this as a goal, from a set of 1301 somatic single nucleotide variants (SNVs) and 240 somatic indels expressed in our GBC samples we predicted high-affinity MHC Class I binding neoantigen peptides (IC50 < 500 nm) (Supplementary Data 11). This resulted in an average of 15 (range 0–51) neoantigens per patient (Fig. 3a). Multiple neoantigens corresponding to mutated *TP53, ELF3, CTNNB1, ERBB2, ARID1A*, and *CDKN2A* were predicted. These genes were mutated in at least 4% of the GBC patients (range 4–17%, *n* = 5–19 from 115 exome/RNA-seq samples; Fig. 3b). Among these, ELF3 had the highest number (*n* = 9) of predicted neoantigens resulting from frame-shift mutations in GBC. A similar trend was observed for TP53 in which 5 of the 9 high affinity HLA binders were frame-shift mutations. Additionally, recurrent mutations in ERBB2 also contributed to potential neoantigenic peptides. The presence of potential antigenic peptides from recurrent somatic mutations in GBC suggests that these peptides can serve as potential common cancer vaccine candidates.

To test the relevance of these predicted neoantigens, we selected 13 mutant peptides and the corresponding wild-type (WT) sequences from ELF3 (6), CTNNB1 (2), ERBB3 (3), and TP53 (2). We tested these peptides for their ability to activate CD8+ T-cells using HLA-matched healthy donor PBMCs. Antigen-specific activation of CD8+ T-cells was assessed by intracellular IFN-γ production using FACS (IFNG-APC (1:100), Biolegend, Cat. No. 502512; Supplementary Fig. 16). Two mutant ERBB2 peptides, S310Y and S310F, and three ELF3 peptides, Y19fs, L73fs and V345fs were found to activate CD8+ T-cells (Fig. 3c, d).

To determine clonotypic changes and activation of CD8+ T-cells in response to the mutant peptides, we perfomed transcriptome-coupled single-cell T-cell receptor (sc-TCR) sequencing. In this experiment, ELF3 Y19fs was expressed as a minigene in dendritic cells and incubated with CD8+ T-cells (see Methods). TCR sequence analysis revealed CD8+ T-cell clonal expansion when incubated with ELF3 Y19fs mutant expressing dendritic cells. However, we did not detect these TCR sequences in the empty vector control treated cells, indicating that they were specific to the ELF3 Y19fs mutant. The clonally expanded T-cells identified contributed to 8% of the total T-cells in the assay (*n* = 670) (Fig. 3e and f). Overlaying the single-cell transcriptome data on the clonally expanded T-cells showed that ELF3 Y19fs induced 3-fold higher levels of IFN-γ transcript in the expanded T-cells compared to empty vector control (Fig. 3g–j). Though some clonal expansion of T-cells was observed in empty vector control, the most frequent clonal sequence represented 2% of the total T-cells (*n* = 178) (Fig. 3e and f**;** Supplementary Data 12). Importantly, the level of IFN-γ was undetectable in these cells (Fig. 3j). Consistent with these findings, the abundance of the CDR3 sequences identified by single-cell sequencing correlated well with those found in the PMBCs treated with the mutant ELF3 peptide (Fig. 3k).

In addition to the immunogenic ELF3 peptides detected in this study, we found TP53 G154V peptide to also be immunogenic as it resulted in a 2.5-fold increase in clonal amplification of T-cells as assessed by TCR sequencing (Supplementary Fig. 17). These findings taken together suggest that the neoantigenic peptides derived from ELF3, ERBB2, and TP53 have the potential for use as cancer vaccines either alone or in combination with checkpoint inhibitors in GBC patients.

MHC I genotype-linked immunoediting is thought to select against specific cancer driver mutations^38^. We assessed if the differences in ELF3 mutation rates between Korean and Indian samples could in part be explained by immunoediting, given the differences in the MHC I alleles in these populations^39^. We performed a neoantigen prediction and binding simulation comparing the specific immunoedited alleles in the two populations (see Methods). We found a slightly higher, albeit significant, percentage of ELF3 neoantigen binding amongst the Indian alleles (43%) when compared to the Korean alleles (41%; OR = 1.1, *n* = 2168 vs *n* = 1060, FET *p* value 0.03) suggesting that immune editing may contribute to the observed regional ELF3 mutation rates.

We performed TCR analysis on bulk RNA-seq data (see Methods and Supplementary Fig. 18) to assess the differences in the T-cell repertoire in ELF3, ERBB2, and TP53 mutated GBC samples. We did not observe significant differences in Shannon Entropy of TCR repertoire diversity between samples carrying the specific neoantigens generating mutations that were tested experimentally in the T-cell activation assay (Supplementary Fig. 18a). However, we did observe a trend for decreased Shannon Entropy of TCR repertoire in ELF3 frameshift mutant samples suggestive of TCR selection. To further power this analysis, we included samples with any frame shift mutation (*n* = 90) versus those without any frameshift (*n* = 25) mutations. Here we observed a significantly lower Shannon entropy (*p* = 0.0008) on the TCR repertoires suggesting that perhaps some TCR selection had occurred in these samples (Supplementary Fig. 18b).

### GBC immune microenvironment

**Fig. 4:**
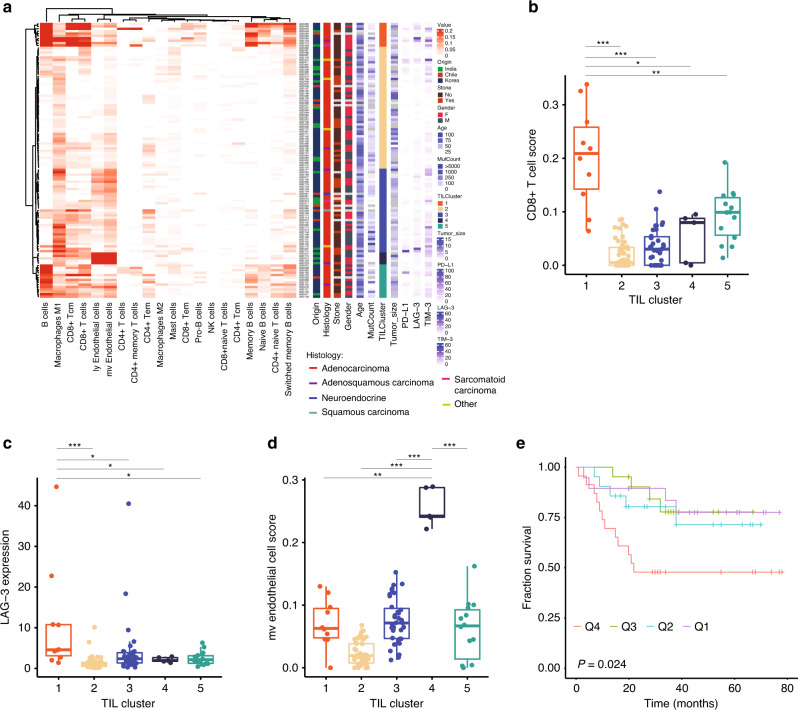
Tumor-infiltrating lymphocyte cluster (TIL) in GBC samples. **a** RNA-seq based determination of cell types and TIL clusters in GBC samples. TIL cluster and their CD8+ T-cell score (**b**; *n* = 115), LAG-3 expression (**c**; *n* = 115), and micro vascular (mv) endothelial cells score (**d**; *n* = 115). Boxes indicate the interquartile range (IQR); center line, median; whiskers, lowest and highest values within 1.5× IQR from the first and third quartiles, respectively. **e** Kaplan–Meier survival plot of GBC samples based on mv endothelial cell score quartiles (*n* = 21 each arm). Log-rank test *p* values are presented for each group (*P ≤ 0.05; **P ≤ 0.01; ***P ≤ 0.001).

Tumor-infiltrating lymphocytes (TILs) and macrophages have been proposed as a prognostic marker in patients with different cancer types^40,41^. They can also affect the efficacy of checkpoint inhibitors^42^. We used RNA-seq data to analyze for presence of TILs and macrophages using the xCell package^43^ (Supplementary Data 13**;** see Methods) and identified 5 distinct clusters (Fig. 4a). Cluster 1 had significantly higher levels of CD8+ T-cells (*p* value <0.05; Fig. 4b) and expression of *LAG-3* (*p* value <0.05), a T-cell suppressor marker (Fig. 4c). *PD-L1* was also higher on average when compared to the other clusters but it was not statistically significant (Supplementary Fig. 19a). Further, the cluster types were not significantly prognostic (Supplementary Fig. 19b; *p* value = 0.45), in part perhaps because of the small number of samples in the clusters. There were no distinctive mutation patterns amongst the five clusters (Supplementary Fig. 19c). Cluster 4 showed a higher level of endothelial cell signature (Fig. 4d). As only two patients in cluster 4 had survival data available, we examined the endothelial score based on quartiles. Patients in the highest endothelial cell quartile had a significant reduction in survival (*p* value = 0.024) (Fig. 4e). Concordant with this the microvascular (mv) gene signature, scores for lymphatic (ly), and endothelial cells are highly correlated (*R*^2^ = 0.9; Fig. 4a).

### KEAP1/NFE2L2 pathway involvement in GBC

**Fig. 5:**
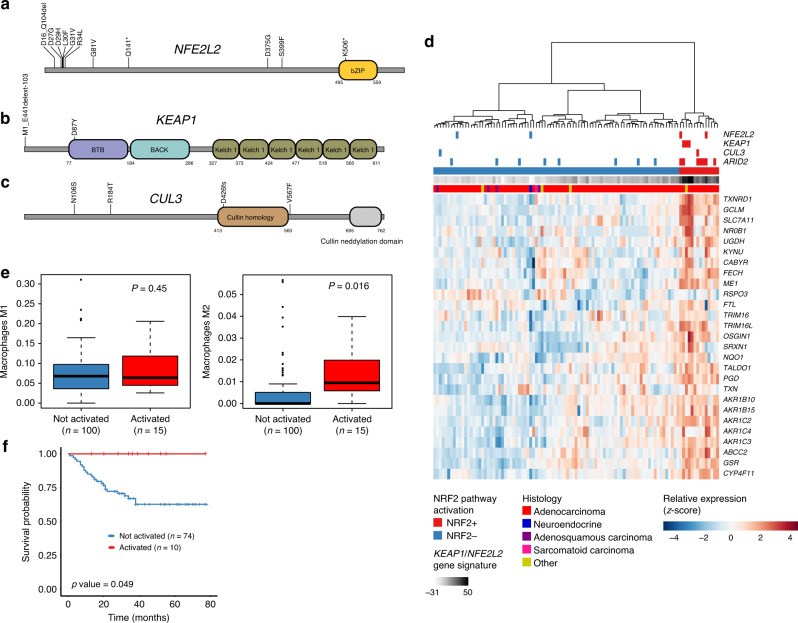
KEAP1/NFE2L2 pathway activation in GBC. Schematics of alterations in NFE2L2 (**a**), KEAP1 (**b**), CUL3 (**c**) from exome, and RNA-seq splicing events (*n* = 166). **d** Hierarchical clustering of GBC samples based on 27 NFE2L2 downstream target genes. Color bars at top of heatmap indicate alterations in NFE2L2, KEAP1, CUL3, ARID2, pathway activation status, and pathway activation score for samples with RNA-seq and paried exome data (*n* = 101). **e** RNA-seq estimated levels for M2 and M1 macrophages for NRF2-/NRF2 + samples (two-tailed Mann–Whitney test). **f** Kaplan–Meier survival plot of NRF2-/NRF2 + samples. Log-rank test *p* values are represented for each group.

KEAP1/NFE2L2, a cellular pathway for sensing and responding to oxidative stress, is frequently mutated in human cancers. We identified several patients with alterations in the transcription factor NFE2L2 (*n* = 11) and its negative regulators KEAP1 (*n* = 3) and CUL3 (*n* = 3) (Fig. 5a–c). Most NFE2L2 alterations (6/11) were found in the N-terminal region required for interaction with KEAP1. Loss-of-function mutations in KEAP1 or activating mutations in NFE2L2 can result in the activation of 27 NFE2L2 downstream target genes, which can be used as a gene signature summarized in a pathway activation score^34^. Application of the gene signature to tumors with available RNA-seq data identified a group of samples that showed elevated expression for most target genes, classifying patients into NRF2+ (score >15) and NRF2- patients. Hierarchical clustering based on the 27 signature genes segregated the two groups (Fig. 5d). Patients with mutations in pathway genes *NFE2L2, KEAP1*, and *CUL3* were overrepresented in the NRF2+ group (*n* = 6/14 NRF2+ vs *n* = 2/87 NRF2- patients, *P* < 6 × 10^–5^, two-sided Fisher’s Exact Test). To search for additional genes that may be involved in pathway activation, we considered significantly mutated GBC genes or known cancer-associated genes recurrently mutated in our data set and tested for overrepresentation among NRF2+ patients. Among 232 genes tested *ARID2* was the most enriched mutated gene (*p* = 0.00074, FDR = 0.13). A recent report^44^ has shown that KEAP1/NFE2L2 pathway activation, which leads to the reduction of reactive oxygen species, may help suppress macrophage inflammation response. Interestingly, we observed no difference in M1 macrophages but found a significantly higher level of M2 macrophages (Fig. 5e; *p* value = 0.016) in NRF2+ samples, consistent with a suppressed macrophage environment. We also found that KEAP1/NFE2L2 pathway activation, using RNA-seq, appears to be a significant prognostic predictor of survival (Fig. 5f; *p* value = 0.049).

### Pathway alterations in GBC

**Fig. 6:**
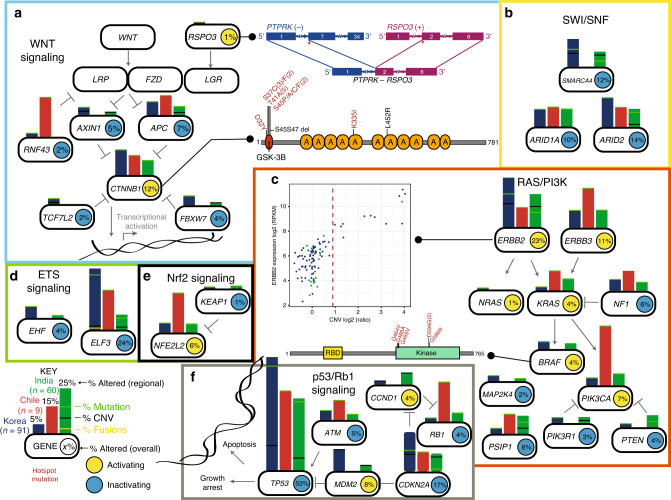
Integrated analysis of pathway alterations observed in GBC. Alterations in the WNT (**a**), SWI/SNF (**b**), RAS/PI3K (**c**), ETS signaling (**d**), Nrf2 Signaling (**e**), and p53/Rb1 (**f**) pathways. Smooth edge rectangles indicate genomically altered genes; bars indicate frequency of genomic alterations by origin (blue, Korea (*n* = 94); red, Chile (*n* = 9); green, India (*n* = 64)). Horizontal lines within bars indicate frequency of mutations (green), fusions (yellow) or copy number loss (black).

We integrated exome, copy number variation and gene fusion data within pathways (Fig. 6a–f) and country of origin. The p53/RB1 pathway was the most commonly altered pathway in GBC (Fig. 6f). The WNT pathway was primarily being driven through activating *CTNNB1* mutations (Fig. 6a) though we also found an activating *RSPO3* fusion. The SWI/SNF pathway had frequent inactivating mutations in *SMARCA4, ARID1A*, and *ARID2* (Fig. 6b). We found many therapeutically actionable mutations in the RAS/PI3K pathway involving frequent alterations involving *ERBB2*, *ERBB3*, *BRAF,* and *PIK3CA* (Fig. 6c). We also found frequent inactivation of the ETS family members *ELF3* and *EHF* (Fig. 6d). Our data demonstrate a role for KEAP1/NFE2L2 pathway activation in GBC (Fig. 6e).

## Discussion

We have performed a comprehensive integrative genomic analysis of 167 gallbladder primary tumors, and 7 GBC cell lines. Also, we have analyzed premalignant gallbladder tissue from 23 cholecystitis cases, 14 gallstones, and 2 gallbladder polyps. We found somatic mutations in cholecystitis that were indicative of a premalignant stage. Our study uncovered a class of hypermutated GBC that carried mutations in mismatch repair genes. We report 25 significantly mutated GBC genes that include several targetable driver genes such as *ERBB2, ERBB3, KRAS, PIK3CA*, and *BRAF*. Importantly, several of the ERBB2 mutations observed are known to be oncogenic and targetable^45^ and patients with such mutations are candidates for targeted HER2 therapy. Analysis of exome and RNA-seq data identified recurrent alterations in KEAP1/NFE2L2 and WNT pathways. Cancer vaccines or checkpoint inhibitors have not been approved for treating gallbladder cancers. We have identified neoantigens from several mutated GBC genes including *ELF3, ERBB2*, and *TP53* and found that they were capable of T-cell activation indicating that they are potential cancer vaccine candidates. Further, we have identified GBC samples with MSI and they likely are candidates for checkpoint inhibitor therapy^46^. Together these findings provide an opportunity for testing immunotherapy in gallbladder cancer.

Overall, our study significantly expands on previous genomic studies providing a comprehensive genomics view of GBCs. Specifically, we identify actionable alterations in over 20% of our cases (Supplementary Fig. 20 and Supplementary Table 4). There are no targeted therapies approved for GBC and current standard-of-care for GBC involves surgery, chemo- and radiation-therapy. Incorporating genomic analysis as part of GBC patient care in the clinic will help improve outcomes through use of approved targeted therapies. Also, the GBC molecular alterations reported in this study and others^5–9^ are an opportunity for development of new therapies.

## Methods

### Samples, DNA and RNA preps

In this study, we analyzed 167 human primary GBC samples as well as 39 non-GBC samples and the corresponding matched normal tissue in most cases using exome-seq, and/or low-pass whole-genome sequencing and/or RNA-seq (Supplementary Table 1). Fresh frozen samples used in the study were obtained from patients undergoing extirpative surgery for GBC. This study was conducted with IRB approval (Pontificia Universidad Católica de Chile IRB, Institutional Human Ethics Committee of Jiwaji University (India) and Seoul National University Hospital IRB (Seoul)) and written patient informed consent. Human tissue samples were de-identified prior to their shipment and analysis and are not considered human subject research under the US Department of Human and Health Services regulations and related guidance (45 CFR Part 46). Basic demographic information for the patient samples in the study, where available, is included in Supplementary Data 1. Tissue processing as well as simultaneous extraction of high-quality genomic DNA and total RNA from the same samples were performed as previously described^47^. The study also included GBC cell lines TGBC24TKB, TGBC2TKB, G-415 (RIKEN Bio Resource Center, Ibaraki, Japan), OCUG-1 (Health Science Research Resources Bank, Osaka, Japan), SNU-308 (Korean Cell Line Bank, Seoul, Korea), GB-d1 (From Dr. Masao Tanaka’s lab, Japan)^48^.

### Exome capture and sequencing

Using the Agilent SureSelect Human All Exome kit (50 Mb), we generated libraries and sequenced them on HiSeq 2500/4000 (Illumina, CA) to generate 2 × 75 bp paired-end data. We obtained a targeted mean coverage of 93x with 93% bases covered at ≥10x (Supplementary Data 2).

### RNA-seq

RNA-seq libraries were generated using TruSeq RNA Sample Preparation v2 kit (Illumina, CA) and sequenced on HiSeq 2500 and HiSeq 4000 to obtain ~63 million 2 × 75 bp paired-end (average) reads per sample.

### Whole-genome sequencing

Low pass whole-genome sequencing (Illumina, CA) data (an average of 2.3x) for tumors and matched normal samples were obtained using whole-genome libraries were prepared according to manufacture’s instructions (Illumina, CA).

### Sequence data processing

We evaluated all sequencing reads for quality using BioConductor ShortRead package^49^. Sample identities were confirmed by comparing exome and RNA-Seq data variants for concordance. We performed an all-against-all sample comparison of germline variants and confirmed the patient tumor-normal pairing.

### Variant calling

Sequencing reads were mapped to UCSC human genome (GRCh38) using BWA software^50^ set to default parameters. Local realignment, duplicate marking, and variant calling of germline variants were performed as described previously^51^. Strelka^52^ was used for somatic variant calling on tumor and its matched normal BAM file. Known germline variants represented in dbSNP Build 131^53^ or found in the ExAC database^54^ at an allele frequency ≥0.1% were filtered out for all samples. The effect of all nonsynonymous somatic mutations on gene function was predicted using PolyPhen^55^. Variants were annotated using Ensembl (release 86). TCGA mutation data used in mutation rate comparison across tumors were retrieved using the CGDSR R package from cBioPortal^56,57^.

### Additional data QC

Sample origins were confirmed using principal component analysis using 5709 common variants^54^ in exomes from this study. Samples were colored according to their known country of origin and clustered using the first and second principal component. Mutant variants with RNA-seq reads count ≥ 1 confirmed their expression. Significantly mutated genes had to have at least one third of their mutations validated by RNA-seq and also have a minimum of half their mutant calls confirmed by the MuTect somatic variant calling algorithm^58^. Recurrently mutated codons that could not be confirmed by RNA-seq were excluded from hotspot analysis, but were retained in the mutation list.

### MSI status determination

MANTIS (Microsatellite Analysis for Normal-Tumor InStability) was used to detect microsatellite instability for all paired GBC samples. Microsatellites within the reference genome (GRCh38) were identified using RepeatFinder, which is included in MANTIS, with default options. MANTIS was run with the options recommended for whole-exome data: -mrq 20.0 -mlq 25.0 -mlc 20 -mrr 1 --threads 8. Samples with a score >0.35 were predicted to be MSI.

### Evaluation of mutations using simulation

A database of all possible nonsynonymous mutations (~70 million) within our exome targets was generated and classified into one of six mutation types, C:G > G:C, C:G > A:T, C:G > T:A, T:A > A:T, T:A > C:G or T:A > G:C. We assessed the functional impact of each mutation using PolyPhen^55^, SIFT^59^, and Condel^60^. Mutations were classified as deleterious when at least two of the three methods employed showed that it had an adverse functional impact. Monte Carlo simulations were performed to assess if the observed ELF mutations differed from randomly generated mutations as described previously^61^.

### Mutational signatures

GBC exome sequence data was analyzed for the frequency of the possible 96 mutation types as described recently^15^. TCGA exome data for 2437 samples from 8 other cancer from SomaticCancerAlterations Bioconductor package and two small cell lung cancer studies^62,63^ was also included in the analysis. We detected a set of six common signatures using Non-Negative Matrix Factorization across the combined data set. Using the mutSignatures (https://cancer.sanger.ac.uk/cosmic/signatures_v2) package^64^ we compared our signatures to that reported in COSMIC^10^ version 2. We also repeated the analysis after removing the MSI samples (Supplementary Fig. 5).

### Mutational significance and hotspot meta-analysis

We evaluated the mutational significance of genes using MuSIC^65^. Given their outlier mutation rate, MSI samples were excluded from this calculation. *Q*-scores were calculated by taking the negative log_10_ of the CRT *q*-values produced by MuSIC and SMGs were selected with a minimum q-score of 1. Germline variants of interest were considered for the following genes: *BRCA1, BRCA2, TP53, MEN1, MLH1, MSH2, MSH6, PMS1*, and *PMS2*. Hotspot meta-analysis was performed as previously described^66^. For the hotspot meta-analysis, we compared GBC mutations to a previous pan-cancer analysis identifying high-confidence recurrent somatic hotspot mutations in cancer^30^. Hotspot mutations within the data set were matched by codon position within a gene.

### RNA-seq data analysis

RNA-seq reads aligned to the human genome version GRCh38 using GSNAP^67^ were used to compute the gene level expression counts. This involved counting the number of reads aligning concordantly within a pair and uniquely to each gene locus using gene models defined by NCBI and Ensembl gene annotations, and RefSeq mRNA database. Variance stabilized expression values for plotting the expression heatmaps were computed using DESeq2^68^. Unsupervised consensus clustering of top 400 most variable genes was performed using the variance stabilized expression values as input to the ConsensusPlus method implemented by the R package ConsensusClusterPlus.

### Identification of transcript alterations

Analysis of splice variants was performed using the R/Bioconductor software package SGSeq (1.8.1)^69^. We performed de novo prediction of gene models from aligned RNA-seq reads for 115 tumors and 4 cholecystitis samples using default parameters. Splice variants were identified from gene models and quantified in terms of FPKM and relative usage PSI (percent spliced-in). PSI estimates with denominator < 10 were set to NA. Splice variants detected in gallbladder samples were also quantified in 9155 normal human tissue samples from the Genotype-Tissue expression (GTEx) project^33^. To identify transcript alterations, we considered splice variants in 835 candidate genes and selected those with FPKM > 2 and PSI > 0.1 in at least one gallbladder sample, and FPKM = 0 in >99.8% of GTEx samples. Identified variants were called in gallbladder samples for which FPKM > 2. FPKM-based criteria were required at both start and end of the splice variant. Alternative starts, ends and retained introns were excluded. Effect on protein-coding potential was assessed with respect to canonical transcript isoforms.

### Low pass whole-genome copy number analysis

The genome was divided in 10 kb bins and the number of reads in each bin provided a count for the genomic bins. This was used to estimate copy number ratio by computing the log2 ratio of the tumor counts with the corresponding normal sample counts and adjusting for total number of reads for each sample. The copy number ratios were then segmented using circular binary segmentation (CBS) and the segments were used to assign a copy number log2 ratio for each gene. Recurrent genomic regions with DNA copy gain and loss were identified using GISTIC2^70^ using log_2_ copy number ratio >0.4 and <−0.4 for gains and losses, respectively.

### Gene fusion detection and validation

Putative fusions were identified using a computational pipeline we have developed called GSTRUCT-fusion^62^. Only fusion events that had at least 4 reads mapping to the fusion junction were included for further consideration. We then further manually curated the fusion results by removing events that are likely false positives.

### Neoantigen prediction and immune editing

The seq2HLA program^71^ was used to assign HLA genotypes based on RNA-seq data using a *P* value cutoff of 0.01. Predicted neoantigens expression was confirmed using the RNA-seq data. The NetMHCcons algorithm from the IEDB software suite^72^ was used to perform predictions on a sliding window of 8–11mers on mutant peptide sequences and the best affinity peptide was chosen as a representative.

We performed immune editing simulations as follows. First, for every Korean and Indian sample, we took HLA genotypes as predicted from the seq2HLA program and recorded the allele on two lists based on origin, HLA-India and HLA-Korea. These lists preserved the observed frequency of HLA alleles observed. Next, we investigated if there were differences in predicted HLA binding affinities based on the HLA alleles observed by origin. To do this, we performed 5000 Monte Carlo Simulations in which we randomly selected a single observed ELF3 mutation as well as a single HLA-Korean allele from their respective list. For each Korean HLA allele and ELF3 mutation we performed neoantigen binding affinity predictions as described above and recorded them as binders (≤500 nm) or non-binders. We then did the same for HLA-India alleles. We then calculated a *p* value for  binders verses non-binders between the two simulations using a two-sided Fisher’s Exact Test.

### Testing neoantigen peptide activity

Briefly, 10 µM wild-type and mutant peptides were incubated with 0.5 million PBMCs in the presence of IL-2 and IL-15 and the incubation mixture was replenished with a fresh batch of peptide-cytokine mix every 3-days. On day-22, PBMCs were harvested and stained for CD3 (0.125 ug/100 ul; Invitrogen Cat. No.12003942;), CD8 (0.125 ug/100 ul; Invitrogen Cat. No. 17008742) and intracellular IFN-γ (Biolegend Cat. No. 502512; dilution 1:100). Antigen-specific activation of T-cells was determined by FACS from the frequency of CD3^+^/CD8^+^ T-cells expressing IFN-γ. Antigen-induced TCR repertoire analysis was performed by subjecting a portion of the PBMC to TCR sequencing using Immuno-SEQ assay (Adaptive Biotechnologies, WA). Unique and shared TCR clones were identified by comparing the TCR repertoire of mutant and WT peptides or DMSO controls. Neoantigens were additionally tested for immunogenicity using a minigene assay  (OncoPept^TM^, MedGenome Inc., CA). Briefly, monocytes prepared from donor PBMCs were differentiated into dendritic cells (DCs)^73^ and transfected with minigenes encoding five tandem sequences of 9-mer mutant peptides or empty vector as control. After 24 h, transfected DCs were co-cultured with 4-fold excess of purified CD8+ T-cells for 10 days. The co-culture was re-stimulated using autologous PBMCs electroporated with the minigene vectors and 48 h post re-stimulation, cells were stained for CD3, CD8, and intracellular IFN-γ to determine antigen-specific activation of IFN-γ. Approximately 10,000 cells from this assay were collected, washed and subjected to single-cell sequencing with immune profiling to determine the gene expression profile in combination with the TCR repertoire as per manufacturer’s instruction (10x Genomics, CA). Sequencing results were evaluated using Loupe Cell and Loupe V(D)J Browsers (10x Genomics, CA) to assess antigen-specific CD8 T cell clonotype induction and their corresponding functional gene expression profiles.

### Estimating cellular content from bulk RNA-seq data

The cellular composition of 115 tumor samples with available RNA-seq data was analyzed using xCell (R package version 1.1.0)^43^. xCell incorporates a novel method to remove dependencies between similar cell types and utilizes gene signatures for over 60 immune and stroma cell types to estimate the enrichment of each cell type in a tissue sample. We then performed hierarchical clustering using the pvclust R package^74^ using the parallel feature and running hclust with method ward.D2 using euclidean distance measurements. We then cut the tree for 5 clusters after visual inspection of the heatmap.

### TCR repertoire analysis

Fastq files were trimmed to remove adapter sequences and low-quality reads using Trimmomatic^75^. The clean fastq files were then analyzed using MiXCR^76^ to identify TCR clonotypes. Based on TCR clones, Shannon index was calculated using R package vegan (https://cran.r-project.org/web/packages/vegan/).

### Actionable alterations in GBC

Actionable alterations were identified by comparing the mutations detected in the study against a list of actionable cancer gene alterations in the OncoKB database (https://www.oncokb.org/actionableGenes).

### Pathway analysis

Mutational significance of genes from the Reactome^77,78^ pathways in the MSigDB v4.0^79^, modified to include *NF2*, was computed using the MuSIC^65^ path-scan program. Integrated analysis of mutation, gene expression, copy number variation and fusion data, limited to samples with alterations, is shown in the quilt plots. Alteration frequencies calculation included all samples that contained data for a given alteration type.

## Supplementary information

Supplementary Information

Descriptions of Additional Supplementary Files

Supplementary Data 1

Supplementary Data 2

Supplementary Data 3

Supplementary Data 4

Supplementary Data 5

Supplementary Data 6

Supplementary Data 7

Supplementary Data 8

Supplementary Data 9

Supplementary Data 10

Supplementary Data 11

Supplementary Data 12

Supplementary Data 13

## Data Availability

The Exome, RNA-Seq and WGS data are available through the European Genome Archive under accession EGAS00001003004 [TCR sequencing/expression data reported in Fig. 3k, e–j can be obtained by contacting the Institutional data access committee gDAC-gbc@gene.com under an MTA that will allow the use of the TCR sequences for non-commercial research studies. All other relevant data are available in the article, supplementary information, or from the corresponding author upon reasonable request.
