# Supplementary material for: Integrated genomic analysis reveals mutated ELF3 as a potential gallbladder cancer vaccine candidate
Source: Nat Commun. 2020 Aug 24;11:4225. doi: 10.1038/s41467-020-17880-4 (PMC7445288; doi:10.1038/s41467-020-17880-4)
Supplement: Supplementary file 1 — Supplementary Information [file 41467_2020_17880_MOESM1_ESM.pdf]

Supplementary Information

Integrated genomic analysis reveals mutated ELF3 as a potential gallbladder cancer vaccine candidate

Pandey et al

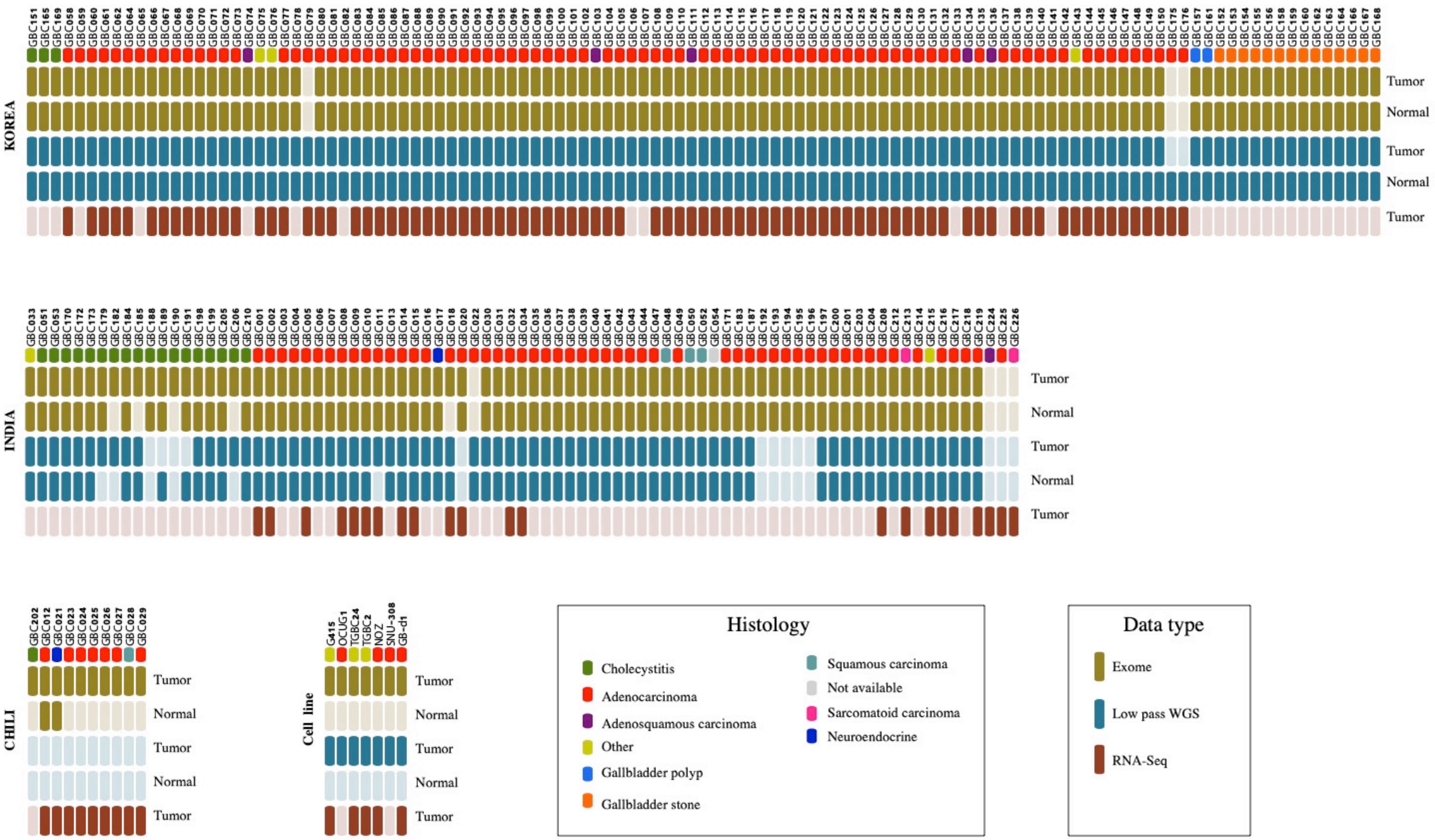

Supplementary Fig. 1. Overview of GBC samples and data types collected.

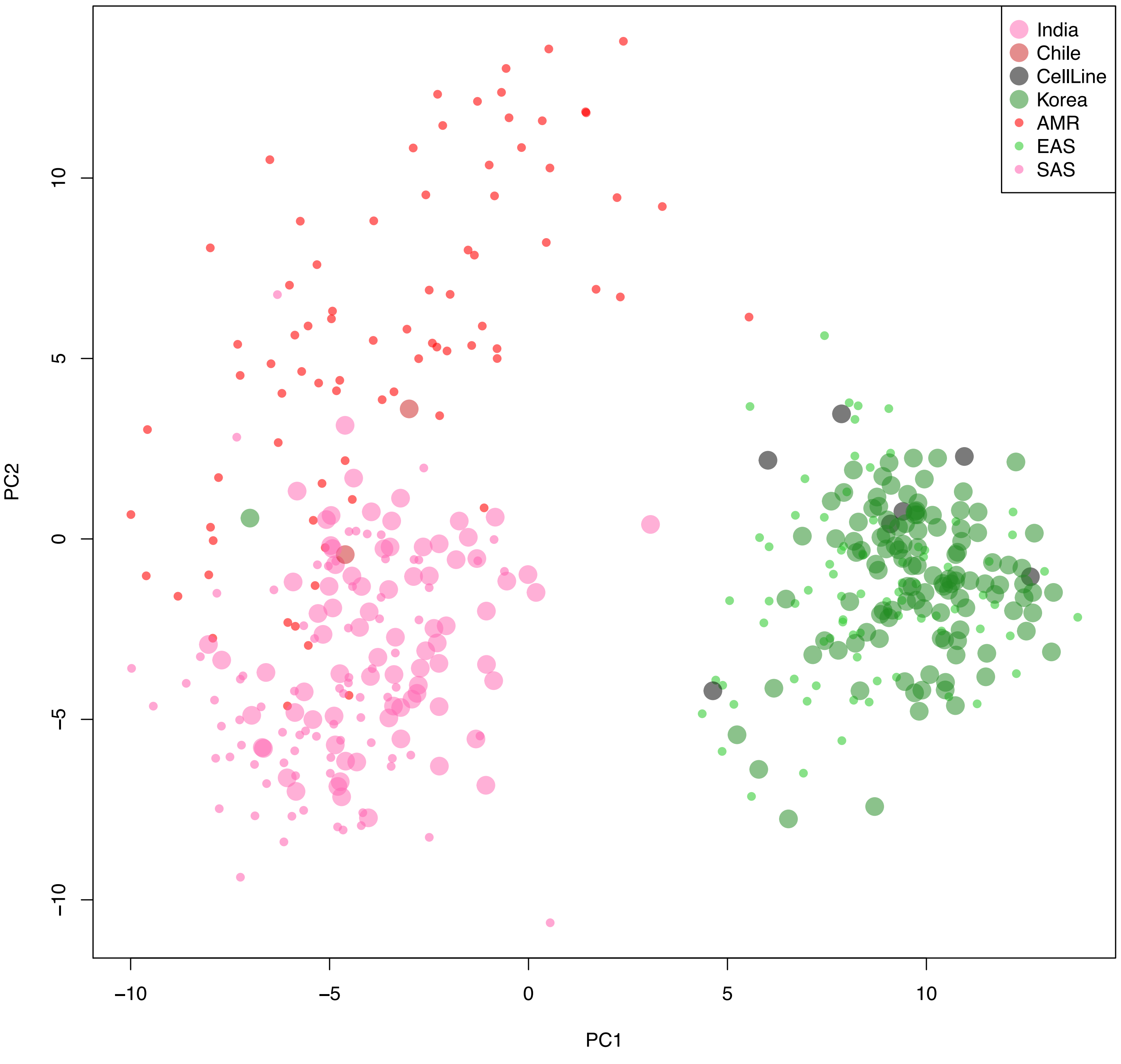

Supplementary Fig. 2. Principal component analysis of germline variants in GBC, GBC cell lines and 1000 genomes data (AMR, EAS and SAS populations).

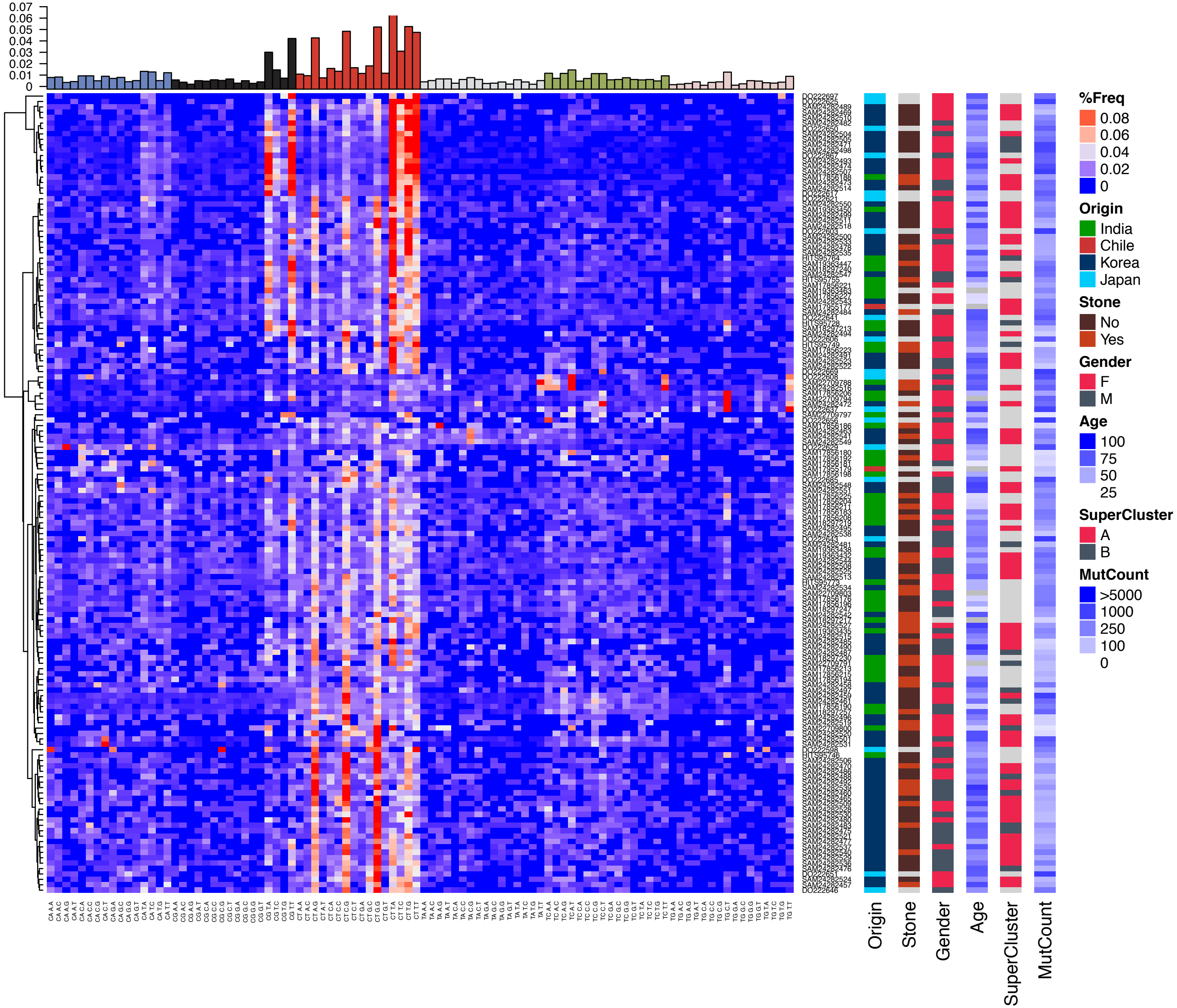

Supplementary Fig. 3. Unsupervised clustering of GBC samples based on observed somatic base substitution frequencies. Each row represents a sample.

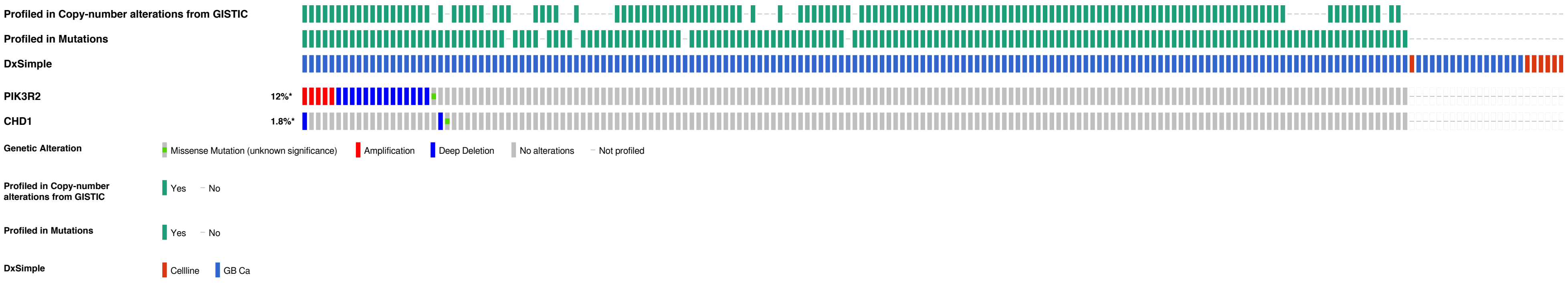

Supplementary Fig. 4. ***PIK3R2*** **and** ***CHD1*** **alterations in GBC and GBC cell lines.**

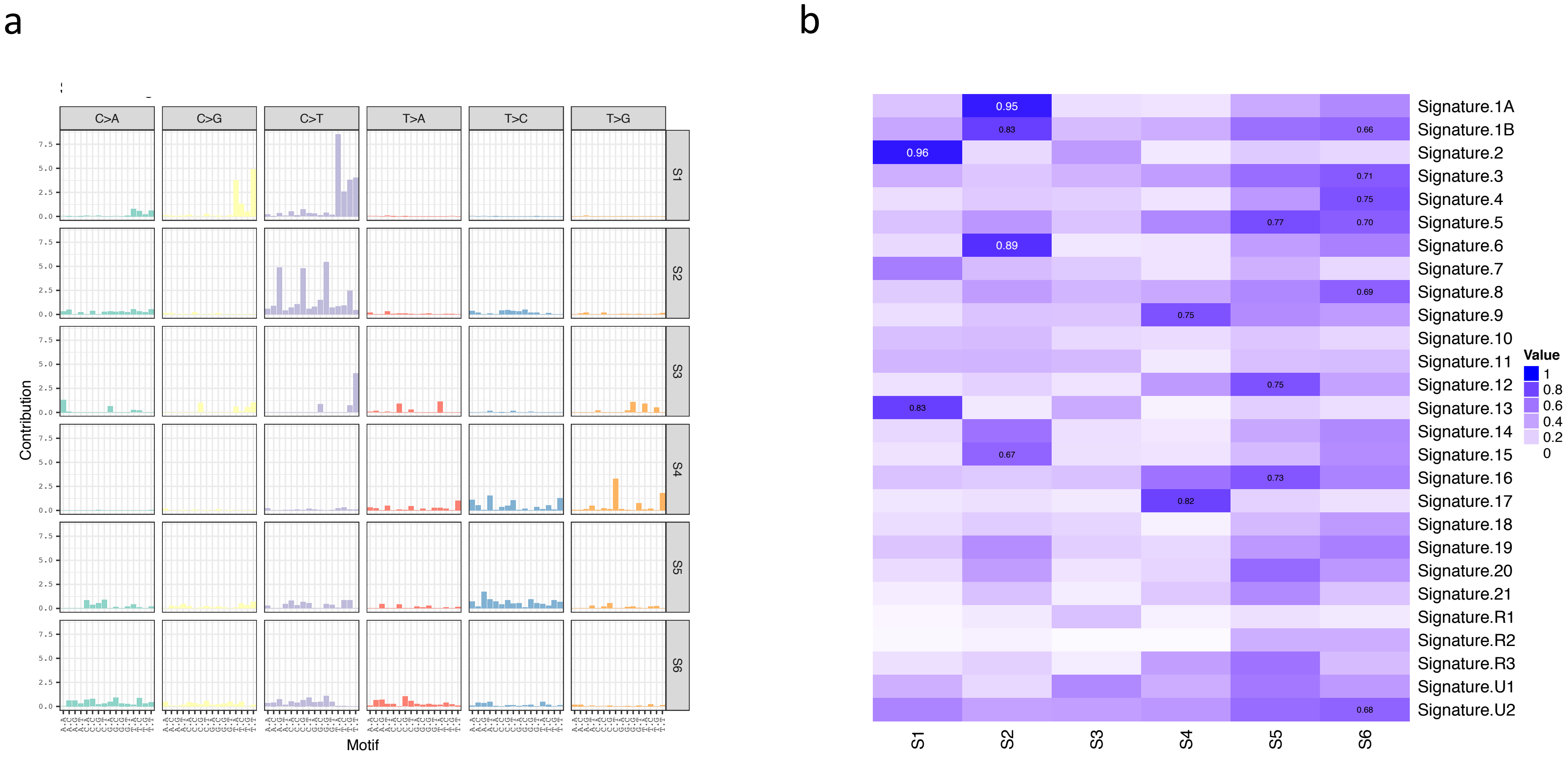

Supplementary Fig. 5. a, Six prominent somatic base substitution signatures found by NMF. **b,** Potential mutational processes responsible for signatures in this study inferred from established signatures shown on the right[1] linked to mutational process.

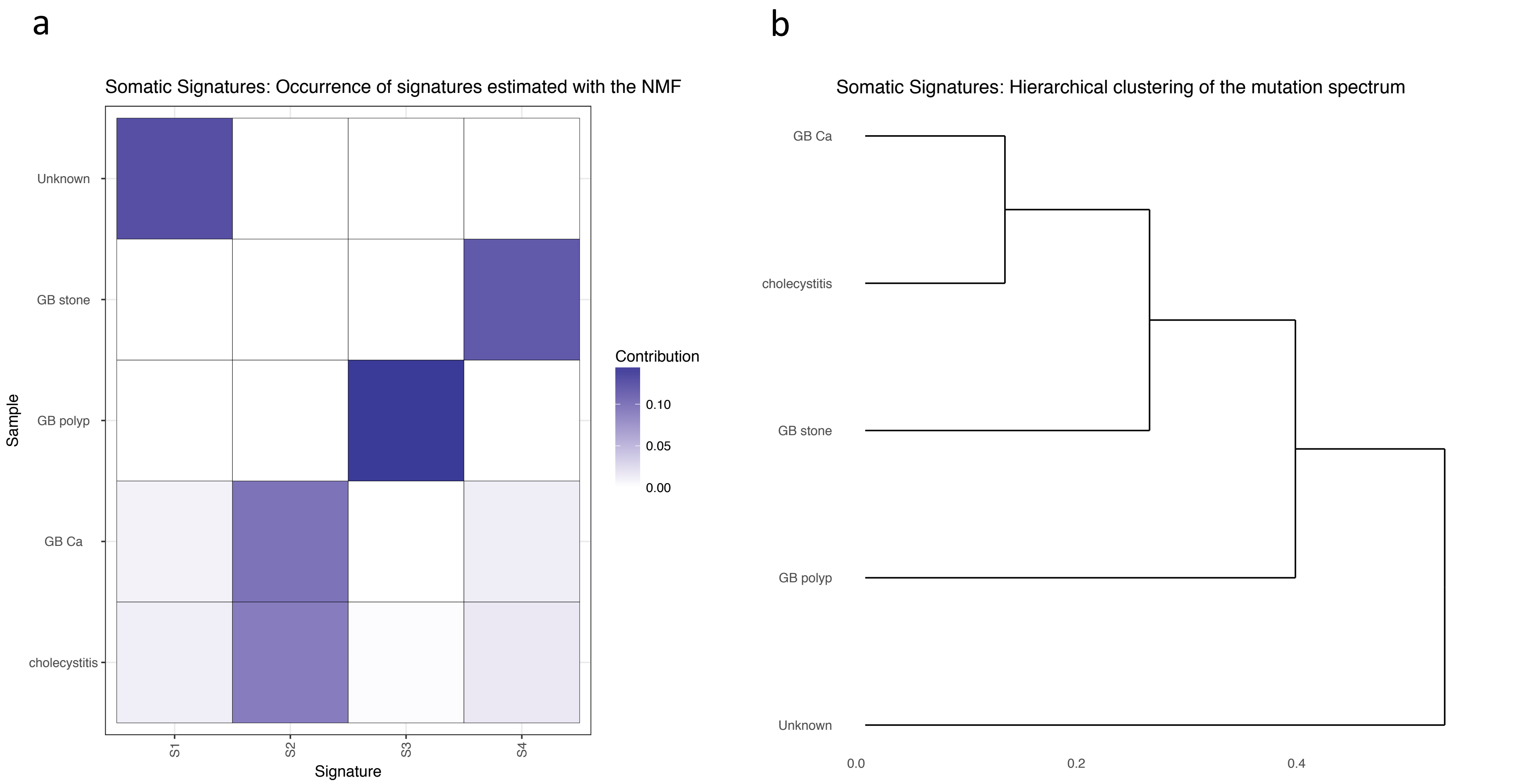

Supplementary Fig. 6. a, Somatic substitution signature-based clustering and **b,** somatic substitution-based hierarchical clustering of GBC, cholecystitis, Gall stone and polyp samples.

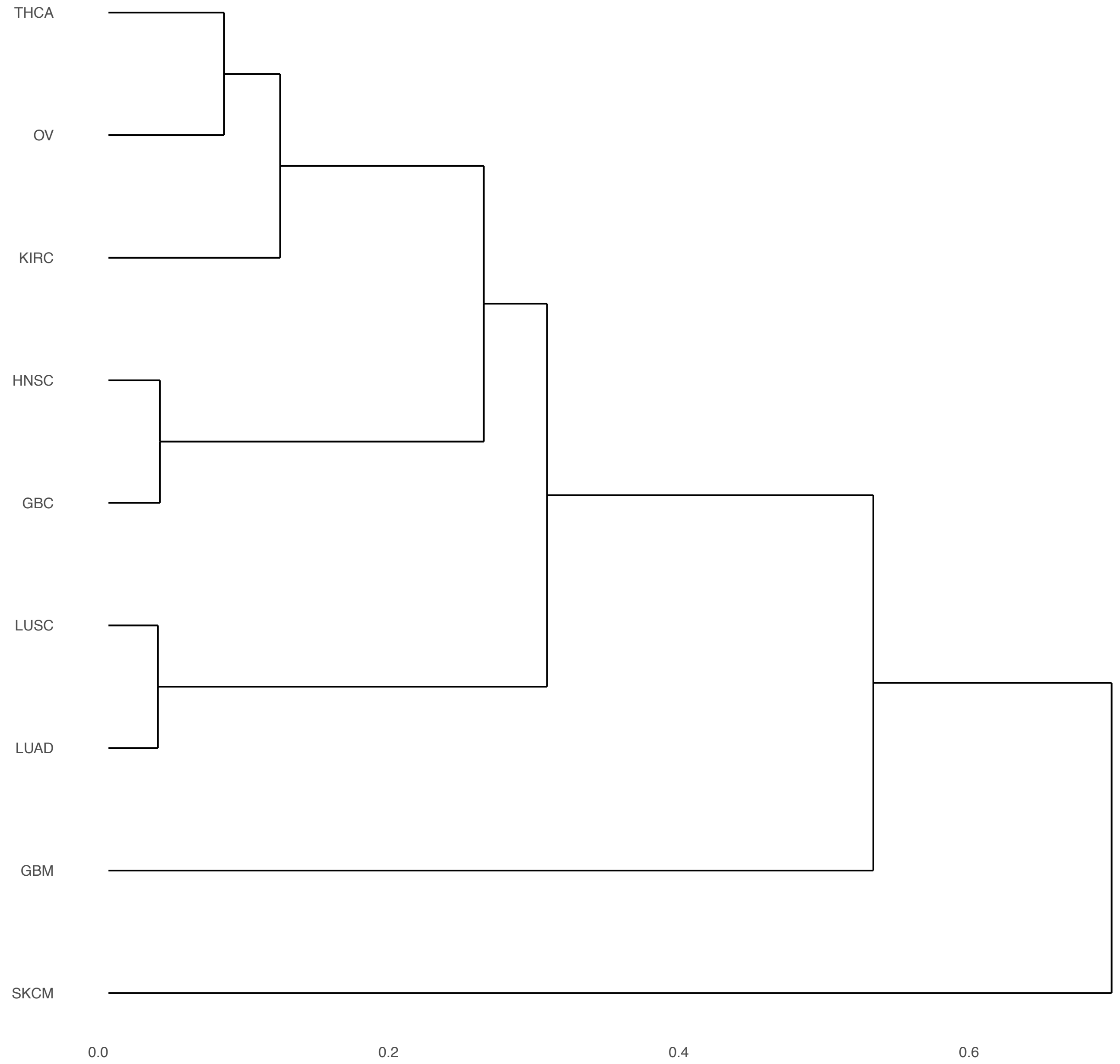

Supplementary Fig. 7. Somatic substitution signature-based clustering of GBC and 8 other non-GBC cancer types

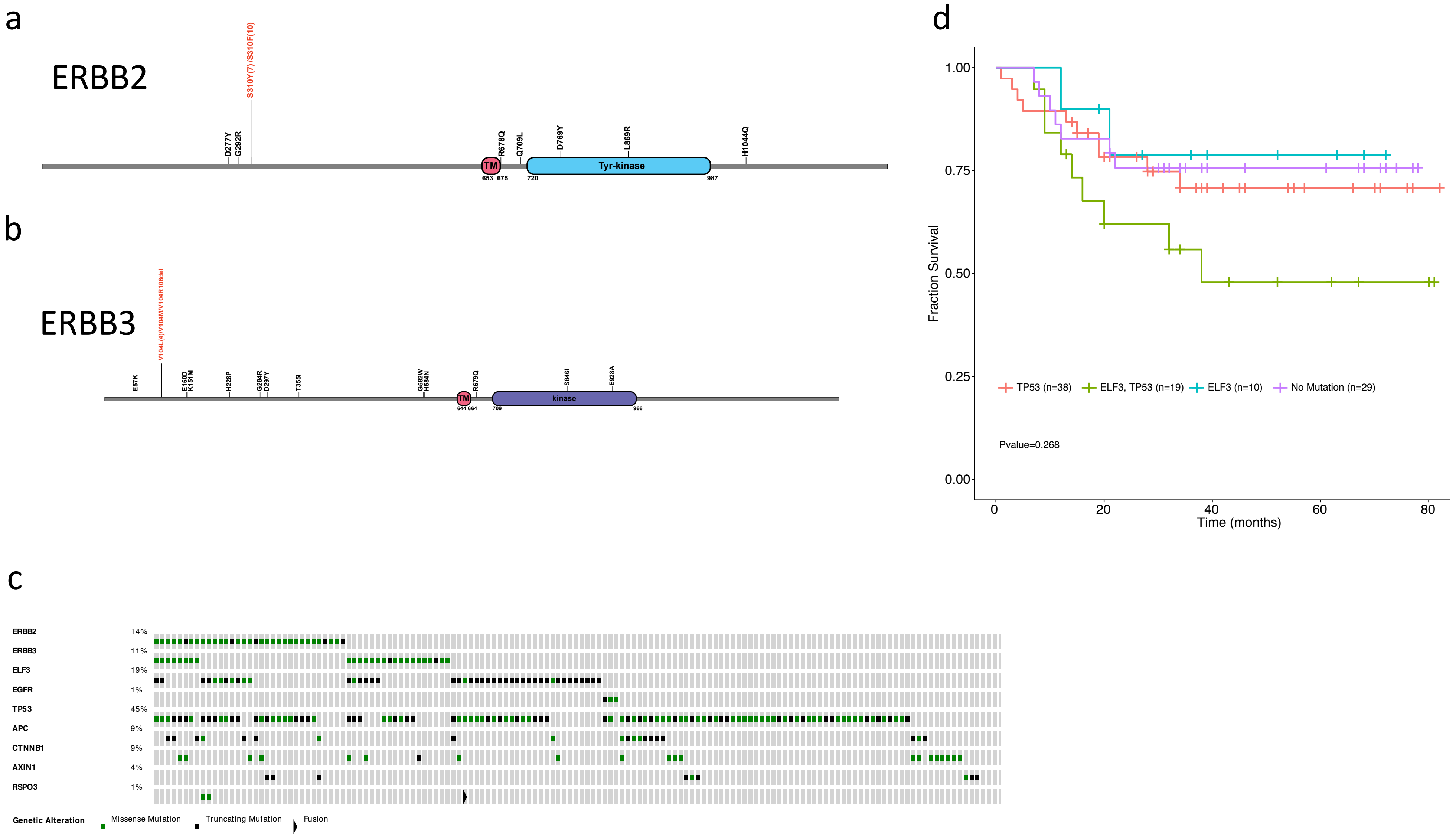

Supplementary Fig. 8. a-b, Protein altering mutation observed in (a) ERBB2 and (b) ERBB3 mutations. c, Quilt plot representing mutations in select indicated genes. Each column represents a sample. d, Kaplan-meier survival plot stratified by samples containing TP53 mutations, containing both TP53 and ELF3 mutations, containing ELF3 mutations and containing neither TP53 or ELF3 mutations. Log-rank test *p*-values are presented for each group

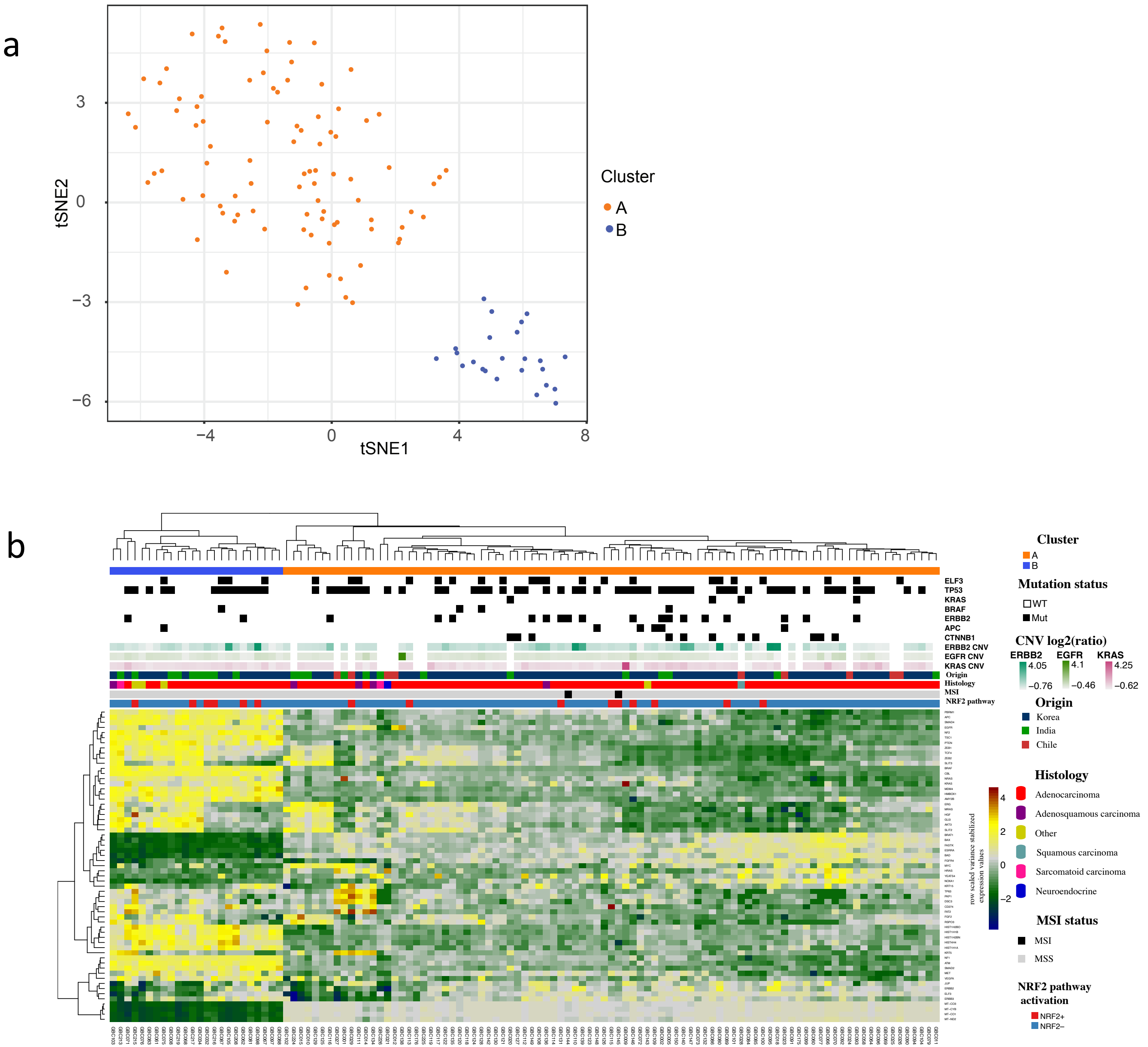

Supplementary Fig. 9. **a,** tSNE analysis of GBC RNA-seq samples **b,** Unsupervised hierarchical clustering of the top 400 most variable genes.

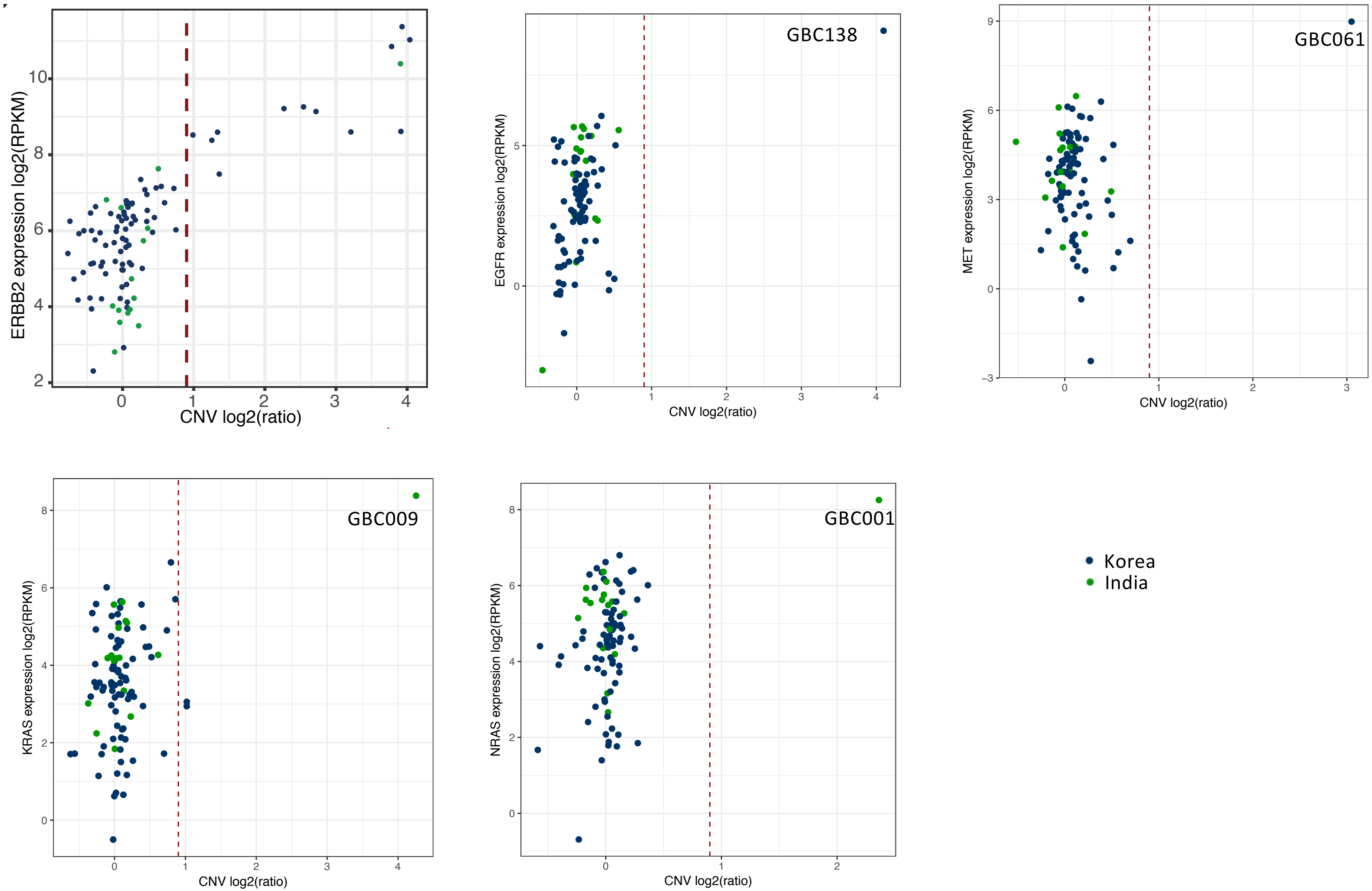

Supplementary Fig. 10. Gene expression vs gene copy-number ratio of indicated amplified genes. The panel showing ERRB2 expression show in main Fig 8 is reproduced here for convenience.

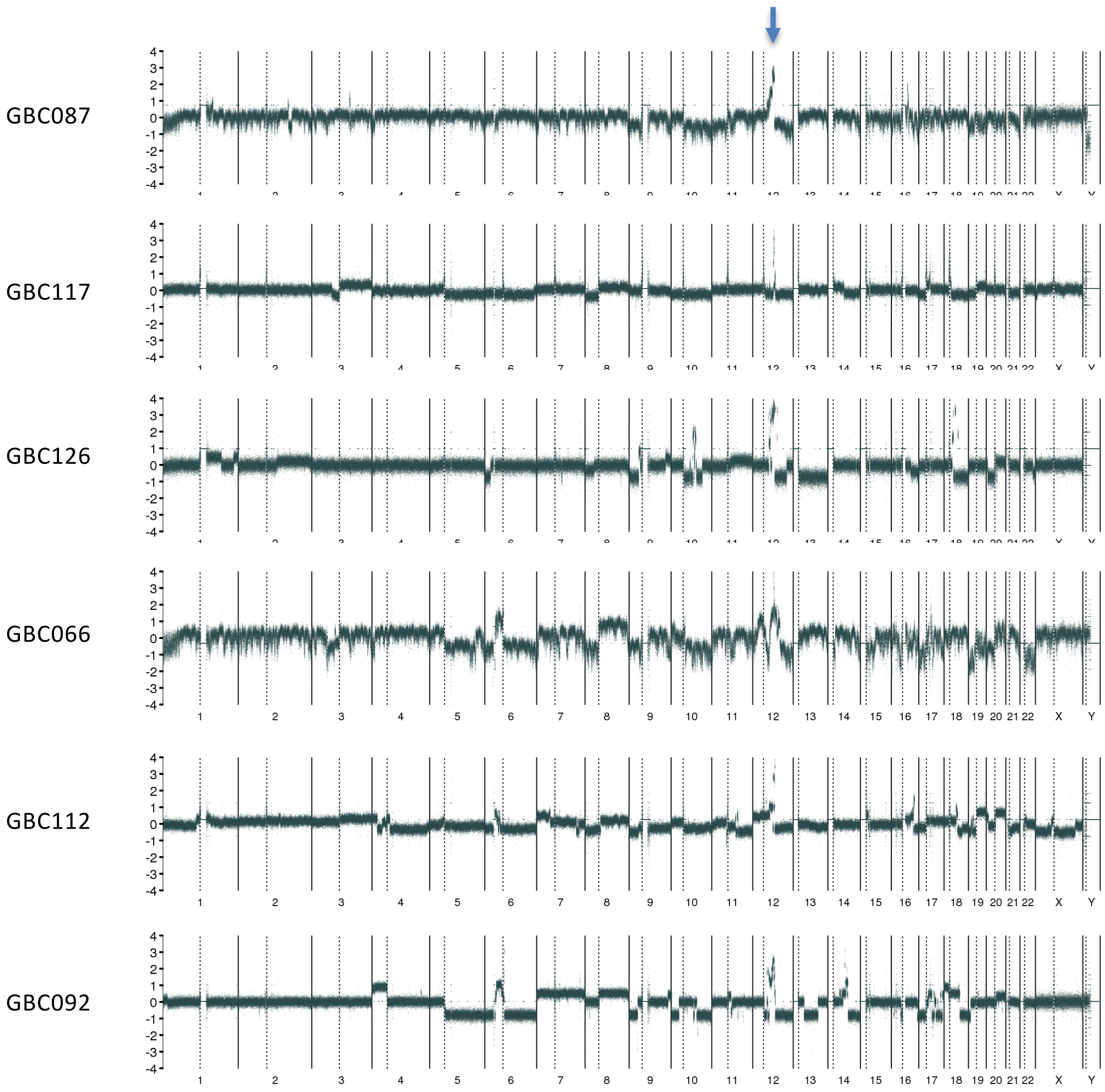

Supplementary Fig. 11: Genome wide copy number plot showing distinct chromosome 12 amplicon present in 6 samples.

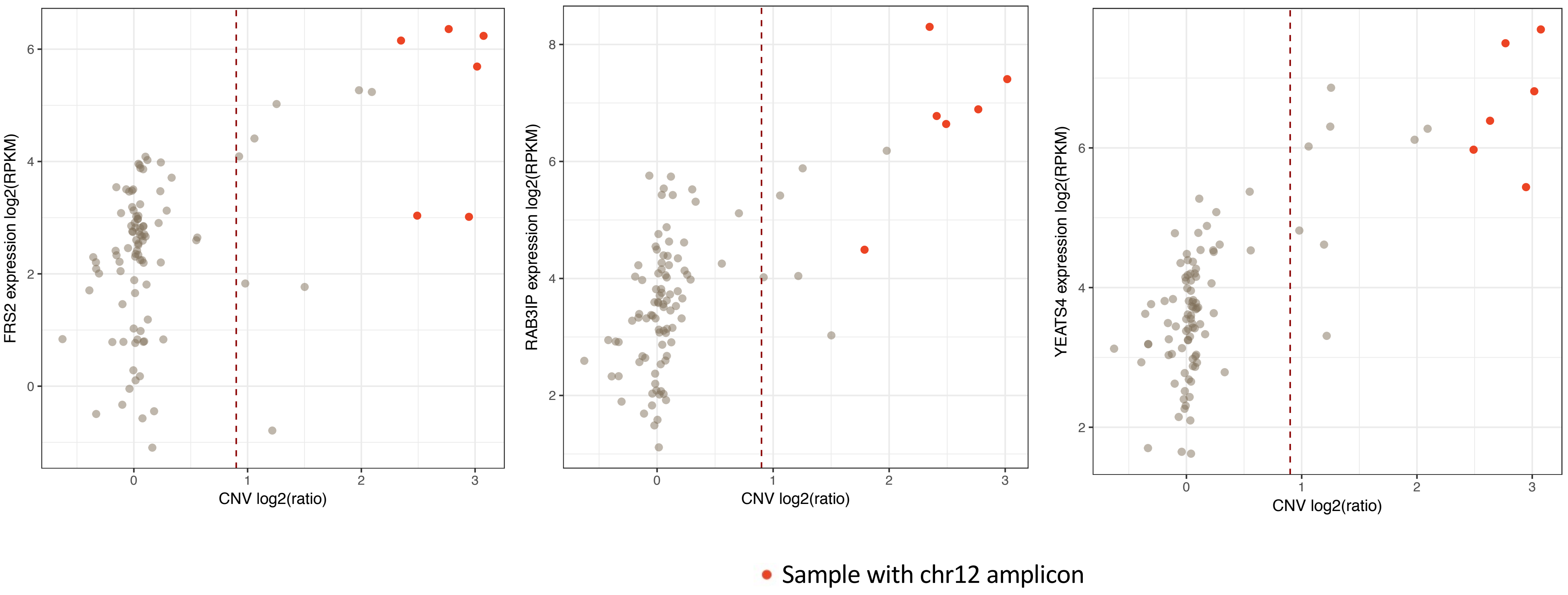

Supplementary Fig. 12: Gene expression vs gene copy-number ratio of FRS2, RAB3IP and YEATS4, located in the amplified region in chromosome 12.

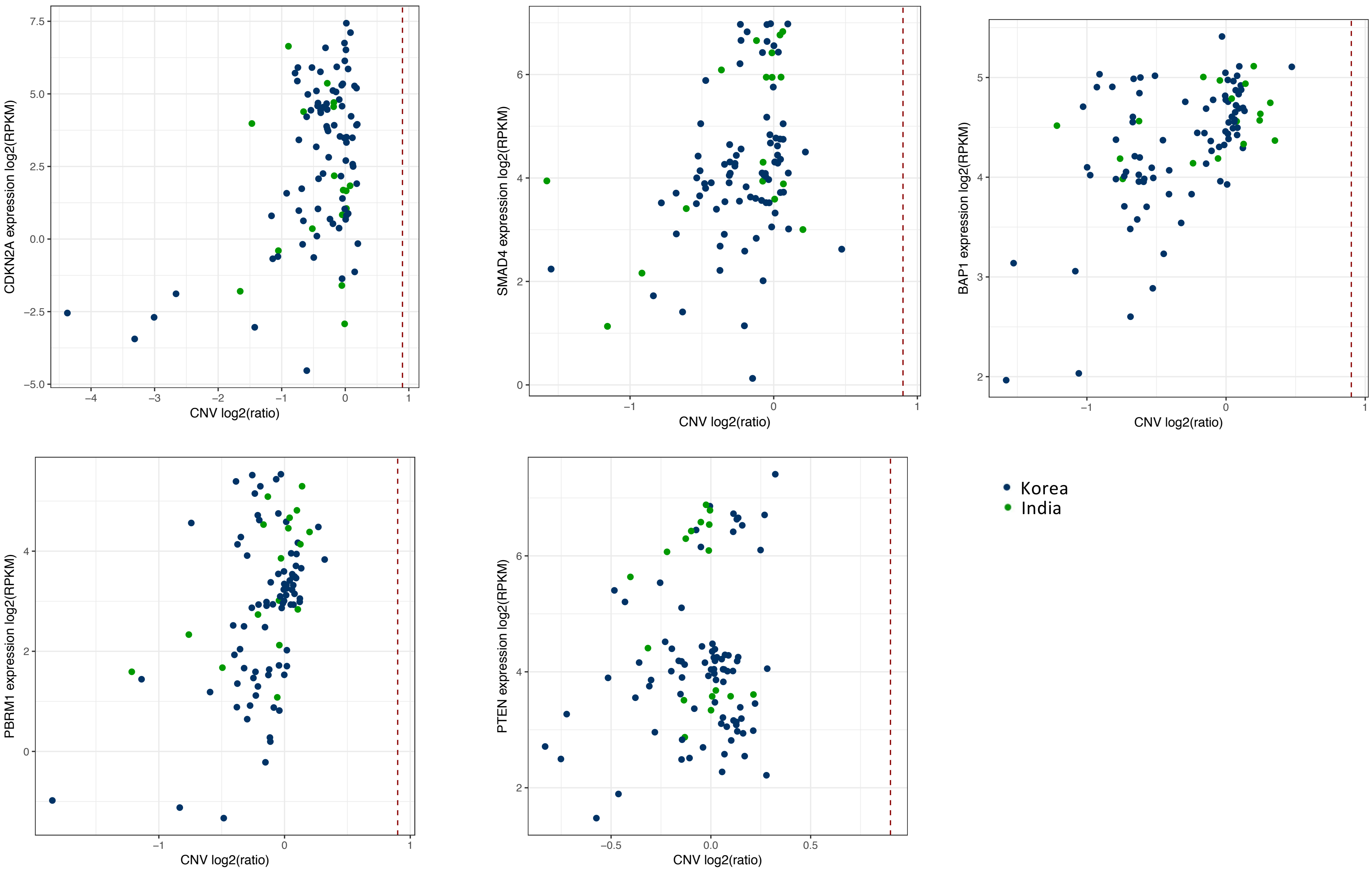

Supplementary Fig. 13: Gene expression vs gene copy number ratio for deleted genes.

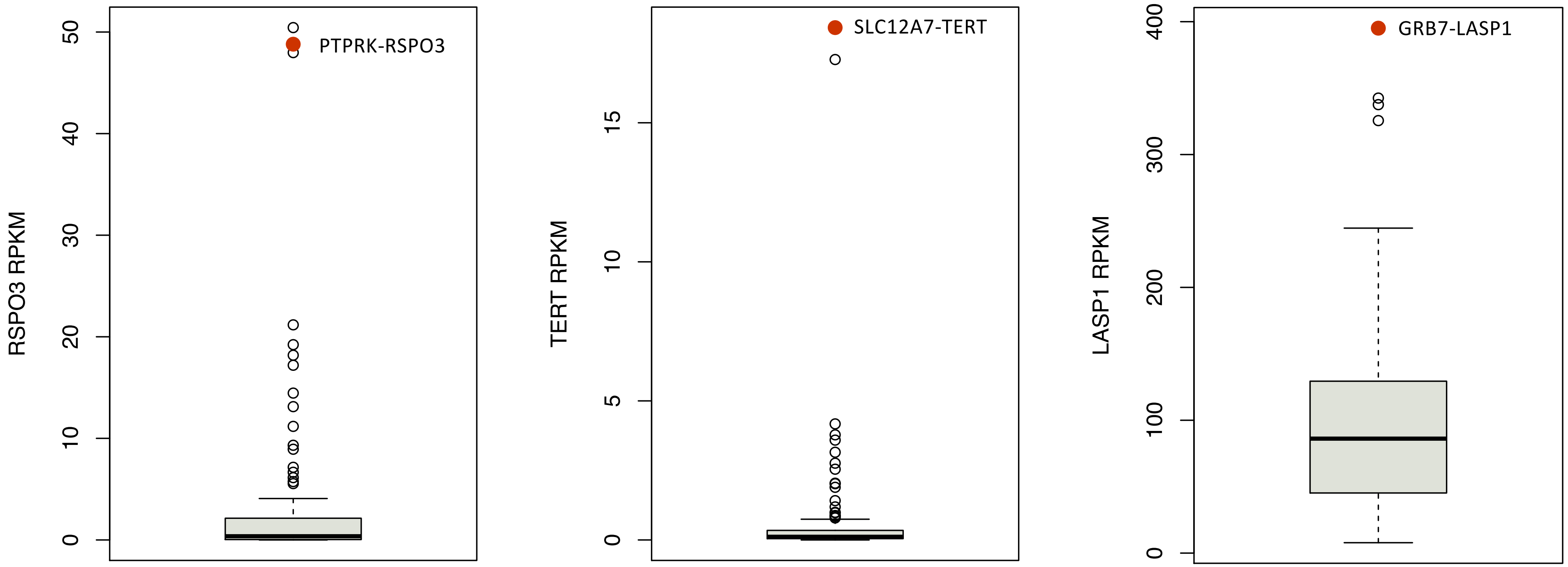

Supplementary Fig. 14: Boxplot of expression levels (RPKM) of select genes involved in gene fusions. Expression values for samples with gene fusion are indicated by a solid red circle. Boxes indicate the interquartile range (IQR); center line, median; whiskers, lowest and highest values within 1.5x IQR from the first and third quartiles, respectively. PTPRK-RSPO3 (n=115) , SLC12A7-TERT (n=115) and GRB-LASP1 (n=115)

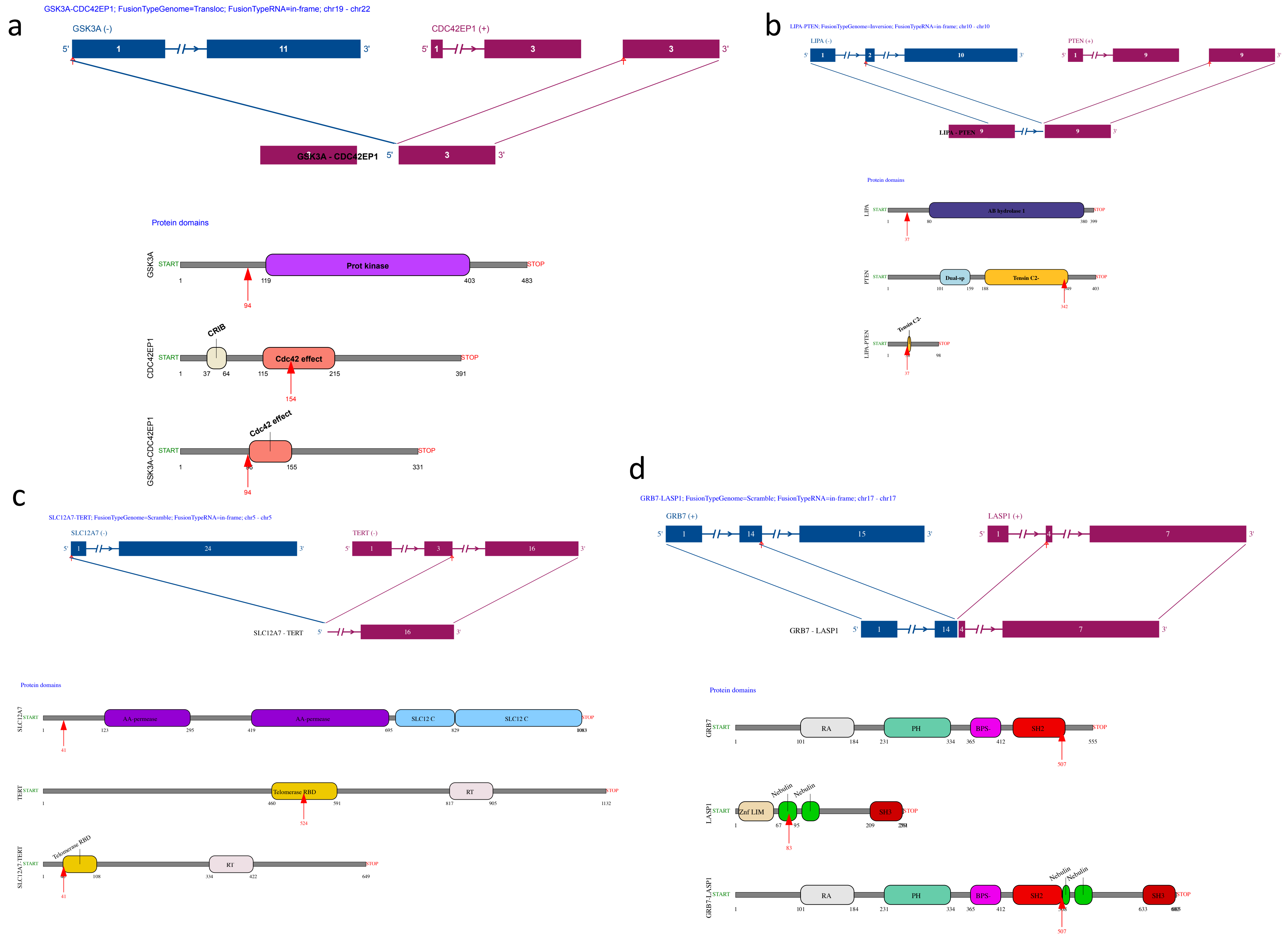

Supplementary Fig. 15. a-d, Schematic representation of gene fusions GSK3A- CDC42EP1 (a), LIPA-PTEN (b), SLC12A7-TERT (c) and GRB7-LASP1 (d)

a

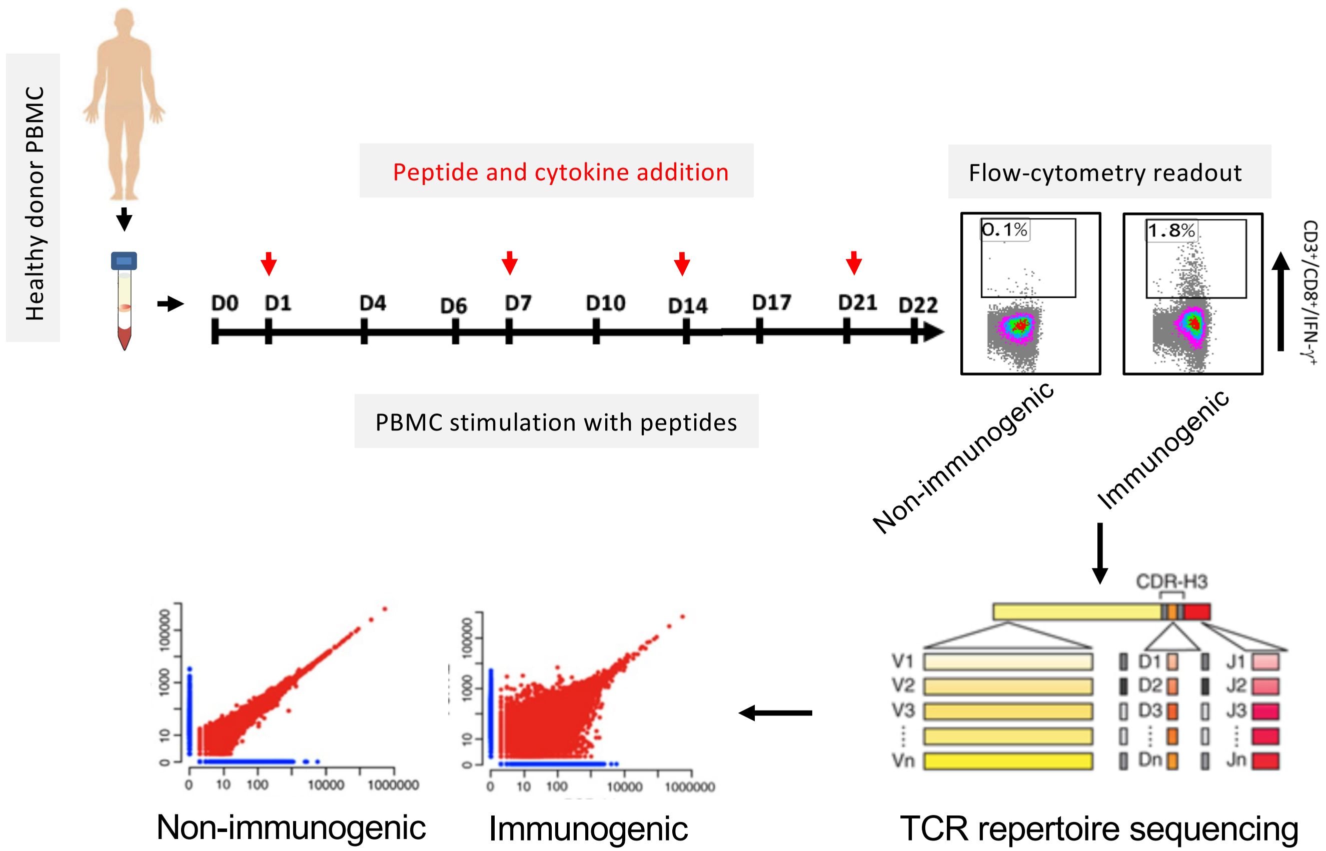

Healthy donor PBMC
Peptide and cytokine addition
D0
D1
D4
D6
D7
D10
D14
D17
D21
D22
PBMC stimulation with peptides
Flow-cytometry readout
0.1%
1.8%
CD3+/CD8+/IFN-γ+
Non-immunogenic
Immunogenic
CDR-H3
V1
V2
V3
Vn
D1
D2
D3
Dn
J1
J2
J3
Jn
Non-immunogenic
Immunogenic
TCR repertoire sequencing

b

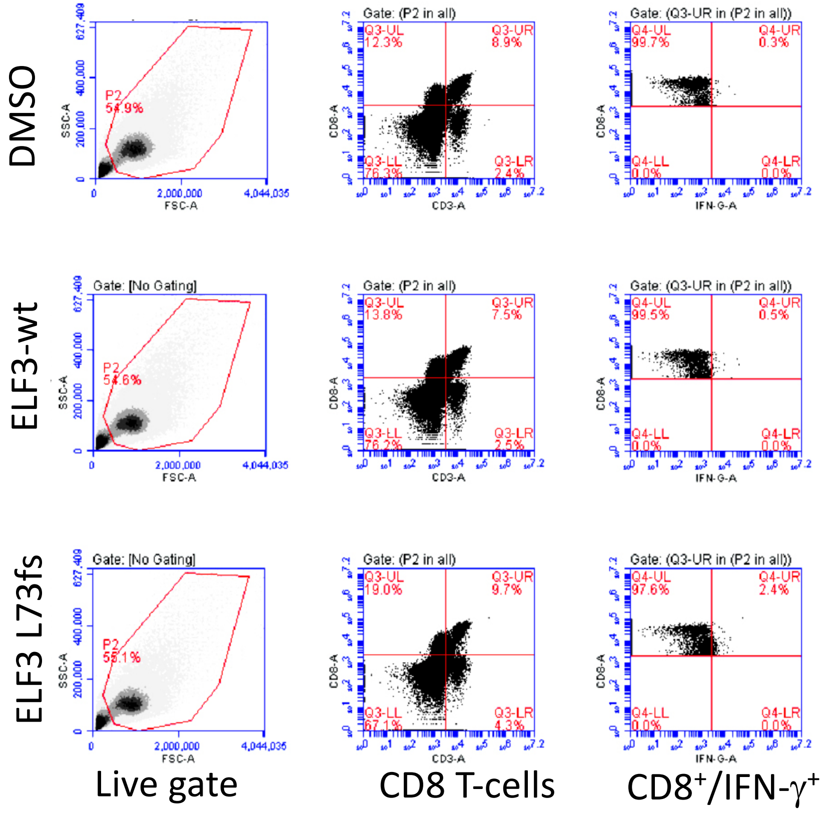

Supplementary Fig. 16. a, Workflow to assess the immunogenicity of the neo-antigen peptides using healthy donor PBMCs. **b,** Gating strategy to identify CD8^{+}/IFN-g^{+} T-cells. Live cells —> CD3^{+}/CD8^{+} —> T-cells CD8 T-cells that are also positive for IFN-g. The same gating strategy was used for identifying activated T-cells in all assays.

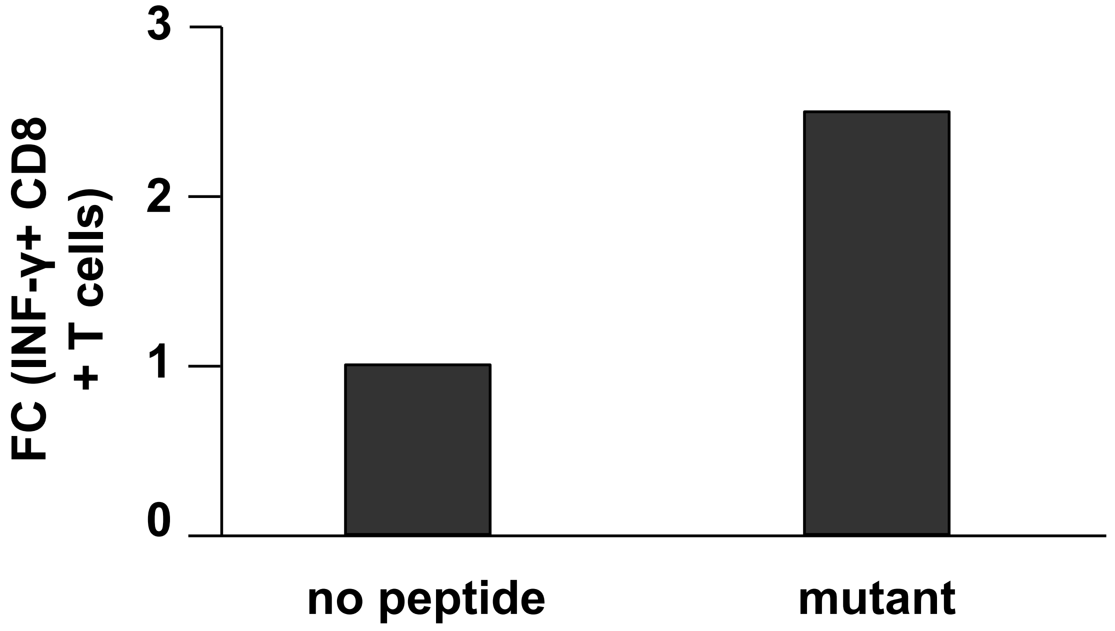

Supplementary Fig. 17. Activation of CD8+ T-cells by TP53 neoantigen peptide measured by INFg secretion (n=1).

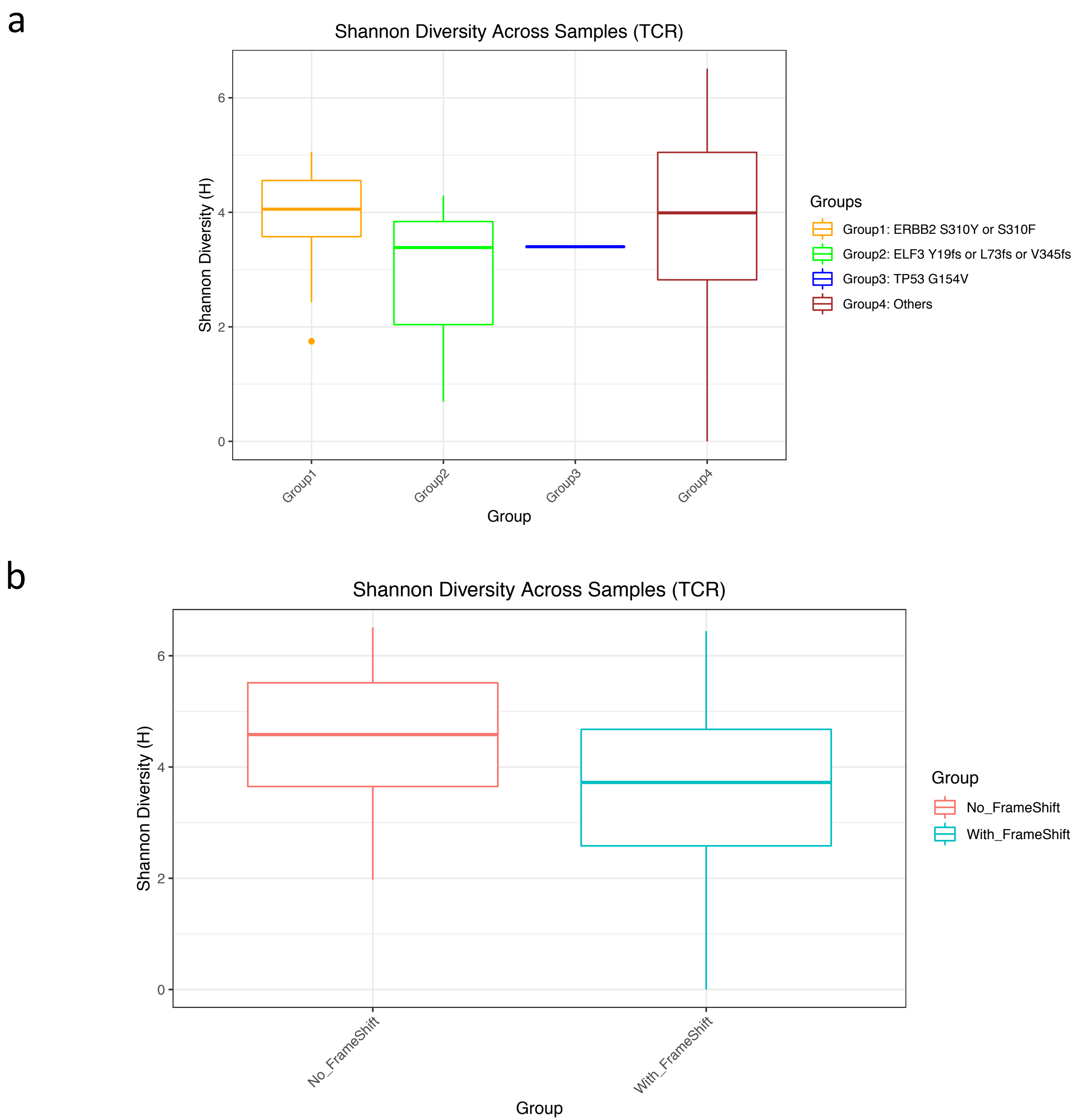

Supplementary Fig. 18. TCR diversity of clonotypes. a, between different neoantigen profile groups (Group1, n =951; Group 2, n=122; Group3, n =39; Group 4, n=13,876) and b, samples with and without frameshift mutations (n=5,591 and 9,413, respectively). Boxes indicate IQR; center line, median'; whiskers, lowest and highest values within 1.5x IQR from the first and third quartiles, respectively

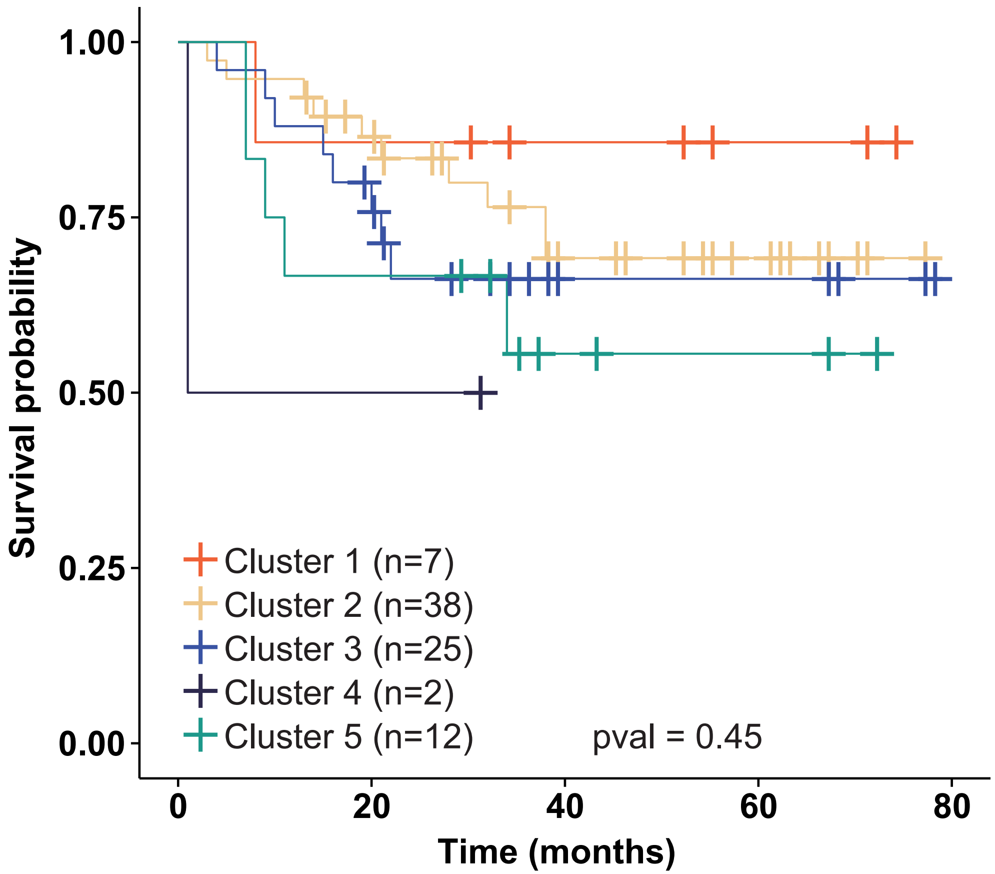

Supplementary Fig. 19. a, PDL-1 expression. b, Kaplan-Meier survival plot of samples stratified by clusters identified using xCell[2].

Actionable Alterations in ERBB2/3, PIK3CA, BRAF

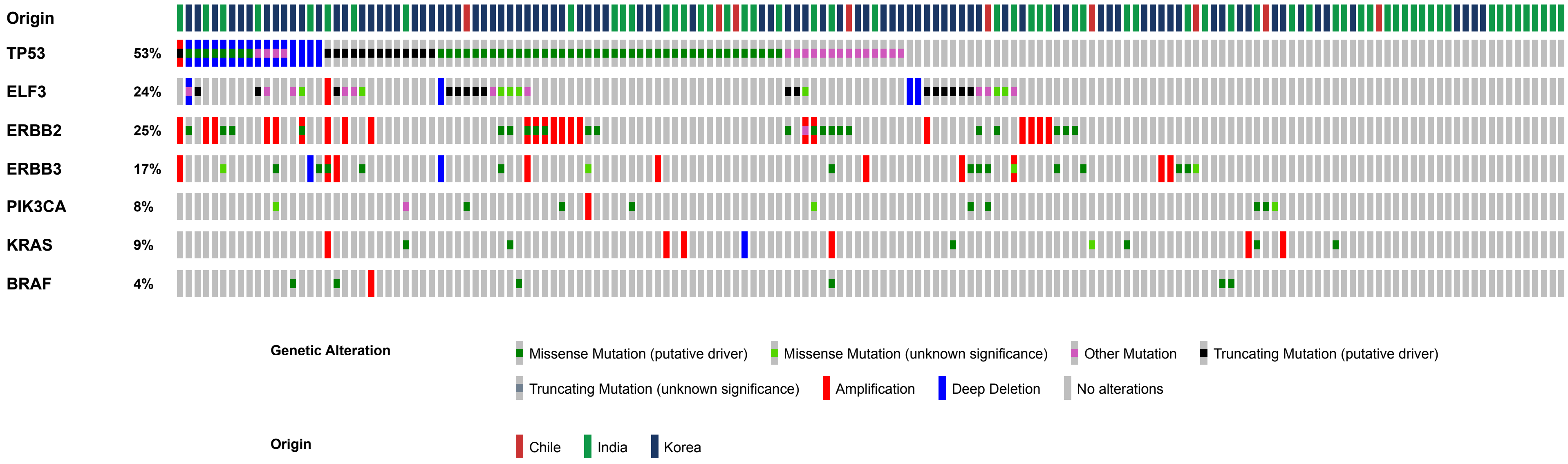

Supplementary Fig. 20. Actionable alterations in GBC.

Supplementary Tables

Supplementary Table 1 - Sample summary

Supplementary Table 2 - Significantly mutated GBC genes

Supplementary Table 3 - Gene fusions

Supplementary Table 4 - Actionable alterations in GBCs

Supplementary Table 1 - Sample Summary

	GBC (n=167)				Choleocystitis* (n=23)				Polyp (n=2)		Stone* (n=14)		Cell line (n=7)		
	India	Korea	Chile	Total	India	Korea	Chile	Total	Korea	Total	Korea	Total	Korea/Japan	Total	All Total
**Total samples**	**64**	**94**	**9**	**167**	**19**	**3**	**1**	**23**	2	**2**	14	**14**	7	**7**	**213**
**WES**	60	91	9	**160**	19	3	1	**23**	2	**2**	14	**14**	7	**7**	**206**
**RNA-seq**	22	84	9	**115**	-	-	-	**-**	-	**-**	-	**-**	5	**5**	**120**
**WGS (~5x)**	54	92	-	**146**	12	3	-	**15**	2	**2**	14	**14**	7	**7**	**184**

Supplementary Table 2 - Significantly mutated GBC genes

Gene	P-value*	False Discovery Rate (FDR)*	# of SNVs	# of Indels	Total # of Mutants	Total # of Samples	Mutation (count)
CTNNB1	0	0	18	1	19	19	D32Y(1), S37C(3), S37F(2), T41A(5), S45S47del(1), S45P(1), S45A(1), S45F(2), S45C(1), K335I(1), L452R(1)
ELF3	0	0	15	22	37	34	Y19fs(1), K56fs(2), L73fs(1), I76fs(1), Y85*(1), splice(4), W138C(1), Q174fs(1), R205fs(1), S207fs(1), G238fs(1), E277fs(1), L283H(1), G301fs(1), L306R(1), W315*(1), K319K320del(1), K320fs(2), N321fs(3), M324fs(1), M324I(1), Y326*(1), E327Q(1), Y336fs(1), K338L342del(1), V345fs(1), D346N(1), F354L(1), G355D(1), W361*(1)
TP53	0	0	67	22	89	80	*394fs(1), Q375*(1), L344P(1), R342*(1), F341C(1), F338C(1), Q331*(2), L308fs(1), splice(4), R306*(1), E286K(1), R282W(1), D281G(1), R280K(4), R280I(1), C277fs(1), R273H(1), R273C(2), S269fs(1), S269N(1), I254T(1), T253A(1), R248Q(1), G245D(1), G245S(1), S241Y(1), S241C(2), C238Y(1), Y234C(1), S227fs(1), Y220C(1), T211H214del(1), R213Q(2), F212fs(1), R209fs(1), D208V(1), L206*(1), Y205H(1), I195N(1), L194R(2), H193R(1), Q192*(2), P177C182del(1), R181C(1), H178fs(1), P177R(1), C176*(1), R175H(7), R175G(1), V173L(1), V173M(2), Y163H(1), I162N(1), R158A159dup(1), V157D(1), G154V(1), V147fs(1), P151S(1), S149fs(1), L145R(1), L137fs(1), N131del(1), S127T(1), W91*(1), A76fs(1), E62fs(1), I50fs(1), E51*(1), P34fs(1), P27fs(1)
ERBB2	1.27E-19	4.82E-16	28	0	28	23	D277Y(1), D277A(1), E286K(1), G292R(1), S310F(6), S310Y(7), R678Q(1), Q709L(1), D769Y(1), V777L(1), I788V(1), V842I(3), L869R(1), H1044Q(1), D1058G(1)
ARID2	3.04E-19	9.62E-16	19	8	27	21	T56fs(1), E282K(1), L381fs(1), S417*(1), P471fs(1), S501P(1), Q526*(1), G638R(1), V757E(1), Q760*(1), S832*(1), S833fs(1), S833C(1), I848fs(1), Q871*(1), Q923*(1), Q995*(1), Q1221*(1), R1272*(1), S1351fs(1), Q1403*(2), S1489*(1), P1582fs(1), Q1711*(1), S1748*(1), D1830fs(1)
ERBB3	7.26E-16	1.97E-12	22	0	22	17	E57K(1), M91I(1), V104M(2), V104L(4), E150D(1), K151M(1), H228P(1), A232V(1), G284R(1), D297Y(1), T355A(1), T355I(1), Y464H(1), G582W(1), H584N(1), S846I(1), E928A(1), R1314H(1)
CDKN2A	4.46E-15	1.06E-11	12	1	13	13	E133*(1), W124*(1), D122H(1), D122N(1), L111P(1), H97Y(1), P94L(2), R88Q(1), G83V(1), P72L(1), splice(1), W34fs(1)
STK11	5.91E-13	1.02E-09	7	4	11	10	K41fs(1), Y60fs(2), K84*(1), splice(2), K175fs(1), K175T(1), P179R(1), Q220*(1), W332*(1)
SMAD4	1.03E-11	1.63E-08	11	2	13	12	R38fs(1), D52fs(1), L57V(1), R135*(1), E330K(1), D355V(1), R361C(1), R361H(1), Q366*(1), R380M(1), splice(1), R445*(1), E538*(1)
ARID1A	1.28E-08	0.0000152	9	7	16	16	Y222*(1), G240fs(1), Y471*(1), Q546fs(1), Q583*(1), S607*(1), V700fs(1), S1153C(1), D1850fs(1), T1892A(1), E2023fs(1), D2133fs(1), R2158*(1), N2220S(1), M2267I(1), S2269fs(1)
EHF	1.39E-08	0.0000155	4	3	7	7	T116S(1), A118fs(1), L125fs(1), M209T(1), K274fs(1), N277S(1), R287Q(1)
KRAS	2.06E-08	0.0000217	7	0	7	7	M189L(1), A146V(1), G13D(1), G12A(3), G12D(1)
NFE2L2	8.05E-08	0.0000728	10	0	10	10	K506*(1), S399F(1), D375G(1), Q141*(1), G81V(1), R34L(1), G31V(1), L30F(1), D29H(1), D27G(1)
PSIP1	0.00000273	0.002036	3	5	8	8	I403fs(1), L377S(1), K286fs(1), E278del(1), K218fs(1), splice(1), V110fs(1), S102*(1)
PIK3CA	0.00000293	0.00206	10	1	11	11	P104T(1), L445F(1), P449I459del(1), E453K(1), L540V(1), E542K(1), E545K(3), E849Q(1), H1047R(1)
ZNF107	0.0000144	0.008788	7	2	9	4	E61fs(2), E254K(1), S297F(1), K304N(1), H415Y(1), E442K(1), S690*(1), T749S(1)
BRAF	0.0000201	0.011589	6	0	6	6	G596R(1), D594G(2), G469V(1), G466A(1), G464V(1)
NF1	0.0000428	0.023204	9	2	11	10	R440*(1), K609*(1), A1197fs(1), Y1784*(1), R1830L(1), K2273*(1), Q2340*(1), K2428fs(1), R2450*(1), F2650Y(1), E2800K(1)
KAT8	0.0000571	0.028919	7	0	7	6	Y90C(1), E102K(1), R140C(1), C316R(1), S348F(1), E350K(1), D355H(1)
APC	0.000141	0.058205	8	5	13	11	R810M(1), S1117Y(1), Q1367*(1), T1556fs(1), M1638fs(1), A1718fs(1), F1838fs(1), E1891Q(1), S1931fs(1), S2110F(1), R2237*(1), P2435A(1), E2639*(1)
CHRM3	0.000176	0.068046	4	3	7	6	K213fs(1), T215A(1), E259K(1), R288Q(1), E432Q(1), R568fs(2)
SMARCA4	0.000187	0.070899	14	0	14	11	L177P(1), S478T(1), R539H(1), L670V(1), Q739*(1), A767V(1), A855V(1), E946K(1), E984K(1), R1157Q(1), R1199Q(1), R1256C(1), G1296S(1), R1307W(1)
ACVR2A	0.000199	0.071748	0	6	6	6	F32fs(1), W159fs(1), V432fs(1), K437fs(3)
MAP2K4	0.000248	0.078347	4	0	4	4	R121Q(1), D300V(1), K365*(1), D397N(1)
HIST1H2AG	0.000245	0.078347	3	0	3	3	A11D(1), L64P(1), E122K(1)

* p-value and FDR for significantly mutated genes were obtained using MuSIC[3]

Supplementary Table 3 - Gene fusions

Sample ID	Gene 5'	Gene 3'	Sample RNA-seq Data ID	# of samples with fusion	Max Read Depth	Distance	Fusion Protein Length	Fusion Type	Fusion effect
GBC009	NDRG1	TG	SAM17856276	1	198	127356	277	Inversion	in-frame
GBC110	GRB7	LASP1	SAM24296847	1	125	847790	685	Scramble	in-frame
GBC079	MPST	CDC42EP1	SAM24296816	1	119	541262	391	Deletion	promoter
GBC009	TSPAN15	HK1	SAM17856276	1	98	141388	1015|928	Scramble	in-frame
GBC128	PTPRK	RSPO3	SAM24296866	1	59	1035784	647	Inversion	in-frame
GBC009	YY1	EVL	SAM17856276	1	35	190071	301|695	Scramble	in-frame
GBC009	TLE3	RACGAP1	SAM17856276	1	33	-	632	Transloc	promoter
GBC009	ASXL1	ELF5	SAM17856276	1	29	-	25|74	Transloc	out-of-frame
GBC020	DNAJC10	GRIK4	SAM17856248	1	27	-	1287	Transloc	in-frame
GBC067	RPTOR	RNF213	SAM24296803	1	21	258045	94	Scramble	out-of-frame
GBC121	SLC12A7	TERT	SAM24296859	1	20	170756	649|158	Scramble	in-frame
GBC109	PDE6B	TRIO	SAM24296846	1	20	-	2021	Transloc	in-frame
GBC215	HIF1A	PRKCH	HITS95755	1	14	252728	80	Scramble	out-of-frame
GBC005	JARID2	GRAP2	SAM19363402	1	9	-	330|29	Transloc	promoter
GBC122	ELF3	PGBD2	SAM24296860	1	8	46901638	592	Deletion	promoter
GBC176	RFT1	ULK4	SAM24296849	1	7	11880764	70	Deletion	in-frame
GBC002	SCARB1	UBC	SAM19363399	1	6	50179	728	Scramble	promoter
GBC135, GE	GSK3A	CDC42EP1	SAM24296873,SAM:	2	6	-	331_331	Transloc	in-frame,in-frame
GBC081	JUP	ACLY	SAM24296818	1	6	102208	425	Scramble	in-frame
GBC213	LIPA	PTEN	HITS95749	1	5	1282251	98	Inversion	in-frame
GBC076	B2M	GNAS	SAM24296813	1	3	-	370	Transloc	in-frame
GBC219	PELI2	MERTK	HITS95731	1	3	-	375	Transloc	in-frame
GBC213	RAP1GAP2	PTEN	HITS95749	1	3	-	375	Transloc	in-frame

Supplementary Table 4. Actionable alterations in GBCs

Sample ID	Actionable alterations	Drugs
GBC002	$ERBB2^{S310Y}$	Neratinib, Ado-Trastuzumab Emtansine
GBC005	$BRAF^{G464V}$; $ERBB2^{G292R}$	Cobimetinib, PLX8394, Neratinib, Ado-Trastuzumab Emtansine
GBC018	$ERBB2^{AMP}$	Lapatinib, Pertuzumab, Trastuzumab, Ado-Trastuzumab Emtansine, Neratinib
GBC020	$KRAS^{G13D}$	Binimetinib, Trametinib, Cobimetinib, Panitumumab, Cetuximab
GBC023	$ERBB2^{V842I, D1058G}$	Neratinib, Ado-Trastuzumab Emtansine
GBC024	$PIK3CA^{H1047R}$	Fulvestrant, Alpelisib, Buparlisib, Taselisib, Copanlisib, GDC-0077, Serabelisib, Taselisib
GBC026	$PIK3CA^{P104T}$	Fulvestrant, Alpelisib, Buparlisib, Taselisib, Copanlisib, GDC-0077, Serabelisib, Taselisib
GBC029	$ELF3^{V345fs}$; $PIK3CA^{E545K}$	Fulvestrant, Alpelisib, Buparlisib, Taselisib, Copanlisib, GDC-0077, Serabelisib, Taselisib
GBC030	$ERBB2^{AMP, L869R}$	Lapatinib, Pertuzumab, Trastuzumab, Ado-Trastuzumab Emtansine, Neratinib
GBC038	$ERBB2^{S310Y}$	Neratinib, Ado-Trastuzumab Emtansine
GBC044	$ERBB2^{AMP}$	Lapatinib, Pertuzumab, Trastuzumab, Ado-Trastuzumab Emtansine, Neratinib
GBC049	$KRAS^{G12A}$	Binimetinib, Trametinib, Cobimetinib, Panitumumab, Cetuximab
GBC062	$ERBB2^{V842I}$	Neratinib, Ado-Trastuzumab Emtansine
GBC065	$ERBB2^{S310F}$	Neratinib, Ado-Trastuzumab Emtansine
GBC066	$ELF3^{Y19fs}$	Potential cancer vaccine candidate
GBC071	$ERBB2^{S310Y, E286K}$	Neratinib, Ado-Trastuzumab Emtansine
GBC080	$ERBB2^{AMP}$	Lapatinib, Pertuzumab, Trastuzumab, Ado-Trastuzumab Emtansine, Neratinib
GBC082	$PIK3CA^{E542K}$	Fulvestrant, Alpelisib, Buparlisib, Taselisib, Copanlisib, GDC-0077, Serabelisib, Taselisib
GBC086	$KRAS^{G12A}$	Binimetinib, Trametinib, Cobimetinib, Panitumumab, Cetuximab
GBC089	$ERBB2^{Q709L}$	Neratinib, Ado-Trastuzumab Emtansine
GBC093	$ERBB2^{S310Y}$; $KRAS^{A146V}$	Neratinib, Ado-Trastuzumab Emtansine, Binimetinib, Trametinib, Cobimetinib, Panitumumab, Cetuximab
GBC095	$ERBB2^{AMP}$	Lapatinib, Pertuzumab, Trastuzumab, Ado-Trastuzumab Emtansine, Neratinib
GBC098	$ERBB2^{AMP}$	Lapatinib, Pertuzumab, Trastuzumab, Ado-Trastuzumab Emtansine, Neratinib
GBC099	$ERBB2^{S310F}$	Neratinib, Ado-Trastuzumab Emtansine
GBC105	$ERBB2^{AMP}$	Lapatinib, Pertuzumab, Trastuzumab, Ado-Trastuzumab Emtansine, Neratinib
GBC107	$ERBB2^{AMP}$; $PIK3CA^{E453K}$	Lapatinib, Pertuzumab, Trastuzumab, Ado-Trastuzumab Emtansine, Neratinib, Fulvestrant, Alpelisib, Buparlisib, Taselisib, Copanlisib, GDC-0077, Serabelisib, Taselisib
GBC108	$ERBB2^{AMP}$	Lapatinib, Pertuzumab, Trastuzumab, Ado-Trastuzumab Emtansine, Neratinib
GBC110	$ERBB2^{AMP}$	Lapatinib, Pertuzumab, Trastuzumab, Ado-Trastuzumab Emtansine, Neratinib
GBC116	$ERBB2^{AMP, D277Y}$	Lapatinib, Pertuzumab, Trastuzumab, Ado-Trastuzumab Emtansine, Neratinib

GBC117	$ERBB2^{S310F}$	Neratinib, Ado-Trastuzumab Emtansine
GBC122	$ERBB2^{AMP}$	Lapatinib, Pertuzumab, Trastuzumab, Ado-Trastuzumab Emtansine, Neratinib
GBC128	$BRAF^{G469V}$	Cobimetinib, PLX8394
GBC131	$ERBB2^{S310F}$	Neratinib, Ado-Trastuzumab Emtansine
GBC132	$ERBB2^{S310F, D277A}$	Neratinib, Ado-Trastuzumab Emtansine
GBC135	$ERBB2^{V777L}$	Neratinib, Ado-Trastuzumab Emtansine
GBC136	$ERBB2^{AMP, D769Y}$	Lapatinib, Pertuzumab, Trastuzumab, Ado-Trastuzumab Emtansine, Neratinib
GBC137	$ERBB2^{AMP}$	Lapatinib, Pertuzumab, Trastuzumab, Ado-Trastuzumab Emtansine, Neratinib
GBC139	$ERBB2^{AMP, S310F}$	Lapatinib, Pertuzumab, Trastuzumab, Ado-Trastuzumab Emtansine, Neratinib
GBC140	$ERBB2^{AMP, S310Y}$	Lapatinib, Pertuzumab, Trastuzumab, Ado-Trastuzumab Emtansine, Neratinib
GBC144	$ERBB2^{S310Y, I788V, H1044Q}$	Neratinib, Ado-Trastuzumab Emtansine
GBC146	$ERBB2^{V842I}$	Neratinib, Ado-Trastuzumab Emtansine
GBC147	$ERBB2^{AMP}$	Lapatinib, Pertuzumab, Trastuzumab, Ado-Trastuzumab Emtansine, Neratinib
GBC149	$ERBB2^{AMP,S310Y}$	Lapatinib, Pertuzumab, Trastuzumab, Ado-Trastuzumab Emtansine, Neratinib
GBC150	$TP53^{G154V}$	Potential cancer vaccine candidate
GBC171	$ERBB2^{AMP}$	Lapatinib, Pertuzumab, Trastuzumab, Ado-Trastuzumab Emtansine, Neratinib
GBC187	$KRAS^{G12D}$; $PIK3CA^{E545K}$	Binimetinib, Trametinib, Cobimetinib, Panitumumab, Cetuximab, Fulvestrant, Alpelisib, Buparlisib, Taselisib, Copanlisib, GDC-0077, Serabelisib, Taselisib
GBC192	$ERBB2^{S310Y}$	Neratinib, Ado-Trastuzumab Emtansine
GBC197	$KRAS^{G12A}$	Binimetinib, Trametinib, Cobimetinib, Panitumumab, Cetuximab
GBC200	$ERBB2^{AMP}$	Lapatinib, Pertuzumab, Trastuzumab, Ado-Trastuzumab Emtansine, Neratinib
GBC203	$ERBB2^{AMP}$	Lapatinib, Pertuzumab, Trastuzumab, Ado-Trastuzumab Emtansine, Neratinib
GBC208	$PIK3CA^{E545K}$	Fulvestrant, Alpelisib, Buparlisib, Taselisib, Copanlisib, GDC-0077, Serabelisib, Taselisib
GBC218	$ERBB2^{AMP}$	Lapatinib, Pertuzumab, Trastuzumab, Ado-Trastuzumab Emtansine, Neratinib

References

1 Alexandrov, L. B., Nik-Zainal, S., Wedge, D. C., Campbell, P. J. & Stratton, M. R. Deciphering signatures of mutational processes operative in human cancer. *Cell reports* **3**, 246-259, doi:10.1016/j.celrep.2012.12.008 (2013).

2 Aran, D., Hu, Z. & Butte, A. J. xCell: digitally portraying the tissue cellular heterogeneity landscape. *Genome Biol* **18**, 220, doi:10.1186/s13059-017-1349-1 (2017).

3 Dees, N. D. et al. MuSiC: identifying mutational significance in cancer genomes. uenome Res. 22, 1589-1598, doi:10.1101/gr.134635.111 (2012).
