# Supplementary material for: Integrated genomic analysis reveals mutated ELF3 as a potential gallbladder cancer vaccine candidate
Source: Nat Commun. 2020 Aug 24;11:4225. doi: 10.1038/s41467-020-17880-4 (PMC7445288; doi:10.1038/s41467-020-17880-4)
Supplement: Supplementary file 2 — Descriptions of Additional Supplementary Files [file 41467_2020_17880_MOESM2_ESM.pdf]

## Descriptions of Additional Supplementary Files

**Supplementary Data 1**

**Description:** Sample information

**Supplementary Data 2**

**Description:** Sample-level exome coverage statistics

**Supplementary Data 3**

**Description:** Somatic mutations in paired GBC samples

**Supplementary Data 4**

**Description:** Putative somatic mutations in GBC samples without matched normal tissue

**Supplementary Data 5**

**Description:** Somatic mutations in Cholecystitis, Gall stone and polyp samples

**Supplementary Data 6**

**Description:** Mutations in GBC cell lines

**Supplementary Data 7**

**Description:** Disease relevant germline mutations in GBC patients

**Supplementary Data 8**

**Description:** Hotspot mutations in GBCs

**Supplementary Data 9**

**Description:** Hotspots identified through meta-analysis

**Supplementary Data 10**

**Description:** Cancer specific neoantigens predicted in GBCs

**Supplementary Data 11**

**Description:** TCR clonal expansion assessment

**Supplementary Data 12**

**Description:** TCR clonal expansion assessment

**Supplementary Data 13**

**Description:** Tumor inflitrating lymphocytic cells
